# Information needs, approaches, and case studies in human health risk communication

**DOI:** 10.1111/risa.14006

**Published:** 2022-09-13

**Authors:** Michael Gochfeld

**Affiliations:** ^1^ Rutgers Biomedical and Health Sciences, Environmental and Occupational Health Sciences Institute and Consortium for Risk Evaluation with Stakeholder Participation (CRESP) Piscataway New Jersey USA

**Keywords:** asbestos risks, cancer risks, credibility, mental models, radiation risks, risk perception

## Abstract

This article uses ten case studies to illustrate the information needs, various communication approaches, and the communicator's role in explaining environmental health risks from a variety of hazards, to a variety of audiences, over time frames from days to years, using in person consultation, lectures, zooms, and email formats. Events often had a long history before the communication began and may have had a long tail afterward. Audiences may be public officials, companies, workers, communities, or individuals. Each individual may have their own understanding or mental model regarding the hazard, exposure, and risk. The communicator's role or intention may be to reassure an audience that has unrealistic exaggerated concerns or fears or to protect a client if the fears are realistic. Or it may be altruistic to inform a complacent audience to take the risks it faces more seriously. Although risk assessment research has advanced the techniques for communicating abstruse probabilities to audiences with low numeracy, in my experience, audiences are unimpressed by precise‐sounding probability numbers, and are more interested in whether exposure is occurring or may occur and how to stop it. Often audiences have reason to be outraged and may be more concerned about punishing wrong doers than about the hazard itself, particularly when the exposure is past and cannot be undone. Thus, there is a difference between discussing the riskiness of a situation (risk communication) and what you are going to do about the situation (risk management). Risk communication is successful when the audience responds as intended, calming down or taking action. These case studies are drawn from a large number of risk communication experiences that I and my Rutgers colleagues have engaged in over the past four decades. Through the 20th century, New Jersey was the most densely industrialized State in United States. New Jersey experienced growth of the chemical and petrochemical industries and the unfortunately profligate disposal of toxic wastes. Having the most Superfund sites of any state is a dubious distinction, but New Jersey also has the most experience in evaluating and responding to these hazards.

## 1 INTRODUCTION

Environmental health risk communication deals with hazards, sources, exposure pathways, and risks from agents or conditions encountered in the home, community, or workplace environment. The importance of environmental risk communication emerged in the framework of risk assessment and risk management embraced by the National Research Council's “Redbook” on *Risk Assessment in the Federal Government* (NRC, 1983). The “Redbook, often recognized as a defining point in the history of environmental risk assessment, did not address risk communication at all.” However, by the end of the decade, NRC's (1989) *Improving Risk Communication* explained that although “good risk communication cannot always be expected to improve a situation, poor risk communication will nearly always make it worse” (NRC, 1989, p. 2). That volume emphasized throughout, the importance of building and maintaining credibility. In the 1990′s the Environmental Protection Agency and other groups stressed the importance of improving risk communication, particularly in the face of uncertainty (Morgan et al., 1990) and low numeracy (Brust‐Renck et al., 2015).

Communicators must understand the many ways in which audiences perceive risks (Slovic et al., 2000), both familiar and esoteric. Kasperson (1986) offered six propositions on public participation to enhance risk communication. Appreciating mental models of risky situations and consequences, helps formulate communications while explaining people's resistance to messages that attack their models (Bostrom et al., 1992; Morgan et al., 2002). Slovic, Fischoff and their colleagues provided risk perception research that clarified factors influencing how the public perceives and responds to hazards and messages about risks. They emphasized several dichotomies which raise or lower perceived riskiness: voluntary versus involuntary; familiar versus unfamiliar; personal or social benefit apparent versus no benefit; able or unable to avoid exposure; and other factors (Slovic et al., 1982; Slovic, 1987). Many risks that people fear are technological, and not all technological risks can be dismissed as safe. “How safe is safe enough?” Fischoff et al. (1978) ask in their psychometric study of perceptions of risks and benefits of various technologies. Fischoff et al. (1993) tied risk messaging to the growing knowledge about how risk perceptions form and influence risky or healthy decisions.

Several books offer a broad range of experiences, practices, recommendations, and warnings for the risk communicator and provide many lessons learned on effective risk communication. For example Covello et al. (1988) published a handbook emphasizing practical approaches to communication. Lundgren 1994, now Lundgren & McMakin, 2018, 6th edition) provided a detailed communication guide. Arvai and River (2013) examined effective (and ineffective) communication over a broad range of environmental hazards. Covello (2021) offers approaches specifically for communicating in a crisis with time constraints and impending danger.

Communicating about radiation, nuclear energy, radioactive waste, and nuclear accidents has been particularly challenging (Covello, 1989). Depending on their age, the public has mental models incorporating images of mushroom clouds, death rays in comic books, Three Mile Island (or more likely the *China Syndrome* movie), Chernobyl and Fukushima. The Fukushima fire, explosion and response were fought “on television” over more than a week, leaving an indelible risk message for many about the risks of nuclear energy (Leong et al., 2014). Effective communication about spent nuclear fuel risks and storage is a continuing need for promoting nuclear energy but is also fraught with uncertainties which have hindered attempts at a national repository. *The Fuel Cycle to Nowhere* (Stewart & Stewart, 2011) chronicles the United States’ inability to establish a geologic repository for spent nuclear fuel. Risk communication about such hazards is hampered by public misinformation and misperception (Covello, 1989), but a frontal assault on misinformation and misperception is not usually effective (Slovic, 1987).

Communicating about energy alternatives (Gochfeld, 2011) and climate change (Bostrom et al., 1994, 2019) likewise has evolved over recent decades. This continues to be challenging, particularly if the risks attributed to nuclear energy are miscalculated and misrepresented (Nuttall & Thomas, 2021). There are ethical concerns about risk communication (Johnson, 1999; Morgan & Lave, 1990). The communicator could be protecting a polluter by downplaying a risk or providing misinformation. The communicator may have a stake in encouraging healthful behaviors, as I potentially did in the Maywood thorium case below (Section 4.8).

This article examines the information needs required for communicating about a variety of environmental health risks from the perspective of an environmental physician. Ten studies cover a range of hazards, a variety of audiences, time frames from days to decades, and various communication channels from in‐person to email, and combinations thereof. Risk communication does not occur in a vacuum and seldom is it simply a statement of fact. A risk communication has a purpose, and the communicator has a role which may be to reassure and reduce unwarranted fear and anxiety or may be to arouse concern and end complacency in the face of real danger. Indeed, in many cases the expert, well‐versed in the underlying science, still must face inadequate information, incompletely characterized risks, fears, and uncertainties.

My audience for this article is anyone involved in communicating about public health or occupational health risks who may benefit from the questions, lessons learned, and pitfalls illustrated by the case studies. My audiences for the communication case studies in this article varied greatly: public officials, school boards, company representatives, physicians, parents, teachers, asbestos workers, community residents, including indigenous peoples.

## 2 BACKGROUND

Risk communications occurs in a wide variety of contexts, through many channels, for a broad range of hazards, over varied temporal and spatial scales, and aimed at a variety of audiences with various “stakes” in the information being communicated. This diversity of contexts is well‐described in Lundgren and McMakin (1998‐2018). The communicator has a desired outcome: a better informed audience, a calmed reassured audience, or an activated aroused, perhaps even alarmed audience when danger is close. As an environmental medicine physician, toxicologist, and risk assessor, I have encountered many situations that required me to communicate about risk to a variety of audiences. The environmental event may be a crisis, sudden in origin with immediate consequences, or a long‐standing event wrapped in a political history and socioeconomic consequences.

I learned about risk communication in the context of a multidisciplinary project aimed at helping journalists appreciate risk and incorporate the risk aspects of a situation in their environmental stories. We used a survey, an analysis of an archive of environmental reporting, and workshops, and discovered to our chagrin, that we had a lot to learn about journalism. Even experienced environmental reporters were not as interested in risk as in other aspects of stories such as responsibility and blame (Sandman et al., 1987). A common challenging scenario in risk communication is a public health official or company representative at a public meeting, trying to reassure a worried (over‐worried) or outraged community (Sandman, 1989) that risks were not as bad as feared or that an apparent cancer cluster was a mere coincidence. By contrast, communication should become a dialog rather than a lecture (Chess et al., 1988; Cho et al., 2015).

Often the risk communication concerns cancer, and the communicator gets bogged down in probabilities and negative exponents. In my experience, when people hear “cancer” they stop listening to details. A patient who learns their diagnosis is “cancer,” does not hear or does not remember the rest of the doctor's message (Parker & Banerjee (2018). Likewise, with communities exposed to a carcinogen. They want to stop the exposure, all of it. They want it “gone” not “covered” and “now” not “later.”

In this context the risk communicator is likely to assume she or he represents a “truth” with a rational, objective appraisal of risks, while the audience is often assumed to be uninformed, misinformed, or worse, incapable of interpreting risk information. Overconfidence is a hazardous trait for both the audience and communicator (Fischoff et al., 1977). A large part of risk communication research focused on communicating the numbers, assuming that if people only understood the numbers, they would not be so fearful. Slovic et al. (2000) showed that people's perceptions of risks differed widely from actual risks, comparing guessed versus actual deaths per year from various causes. Comparative risk graphs were aimed at comparing unknown, unfamiliar hazards with more familiar risks (Lungdren & McMakin, 1998, p. 196), such as drowning or smoking a cigarette each day. This was a potential trap in an audience of nonswimmers and nonsmokers. Various creative techniques have been developed to assist risk communication, for example, focusing on the probably long time intervals between low probability events (Weinstein et al., 1996) or the risk of dying from an airplane crashing into your house (Goldstein et al., 1992)

Risk assessment is a highly quantitative enterprise; hence risk communication often involves some numerical or probabilistic presentation. When audiences responded negatively to risk communications, objecting to miniscule risks, it was assumed they did not understand the numbers. Many attempts were made to improve the graphics (Weinstein et al., 1994). One approach shows 1000 figures in neat rows representing 1000 people, with one individual shown in a color, intended to convey a miniscule 1 in a thousand risk. “That's me,” one person responded on seeing the graphic. It has been shown that some people see themselves in the numerator while the communication message is that they are part of the huge, safe, denominator (Lippman‐Hand & Fraser, 1979). I call this the “where's Waldo fallacy,” referring to a tendency for people to see themselves in the one unique figure, missing the point of the graphic. The NRC (1989) *Improving Risk Communication* explained the challenges clearly and offered good advice in a partly mistaken belief that poor numeracy underlay people's unwillingness to accept reassurance. As the mayor of a north Jersey town said at a public meeting, responding to an intended reassurance about a one in a million risk of something—“I don't care if the risk is one in a billion, I don't want it in my town.”

Sandman (1989) explained that the common definition of risk: Risk = Hazard × exposure works on paper, but not in front of an angry crowd where “Risk = Hazard + Outrage.” The audience does not necessarily harbor a flawed mental model that can be corrected with numbers and “facts.” If the audience is outraged they want to hear who is going to jail and will be resistant to messages that downplay their risk. This is illustrated in *The Unfinished School* case (4.10) at the end of this article.

Risk communication took on a more central role with publication of the *Presidential/Congressional Commission on Risk Assessment and Risk Management* report (PCCRARM, 1997) which offered an entirely revised paradigm of risk management. Risk assessment was now depicted as an integral part of risk management, and stakeholders were at the center of the entire model, requiring two‐way risk communication at every step from planning the assessment to evaluating the management. This made effective communication an essential component of successful risk management (Goldstein et al., 2000, 2005; PCCRARM, 1997).

The risk communication task would be much easier if the communication could begin at the beginning of an event and become part of a developing crisis. But that would be a luxury. Crises typically start last week or last month. The communicator is at a disadvantage if they do not know the history. You might be prepared to address health‐related questions such as “will I get cancer?” when the audience really wants to know: Who's at fault? Is it criminal? Can it be fixed? Who pays? Who should resign? Go to jail? Or perhaps: “Would you let your mother live here?” The importance of recognizing and integrating the moral or culpability component as an additional dimension of risk was emphasized by Gardoni and Murphy (2014) who wrote “A risk is ranked higher on the scale the larger the consequences, the greater the probability, and the more morally culpable the source.”

Risk communication is handicapped by prevailing uncertainty (Morgan et. al., 1990) regarding environmental toxicology and exposure assessment. In the 2000–2010 decade, risk assessment confronted uncertainty in various ways that were not always easy for risk communicators (NRC, 2009). The communicator may find it difficult to say, “I don't know,” less difficult to say, “we don't know,” and perhaps, “no one knows” whether it is bad for you. For example, trichloroethylene (TCE), one of the commonest industrial solvents in Superfund sites, was treated as only a possible carcinogen prior to 2011, at which point EPA changed its message, declaring it a known human carcinogen (Chiu et al., 2013) thereby requiring revisiting many earlier risk assessments for virtually every toxic waste site and rewriting risk communication messages (Gochfeld et al., 2022)

Often at a public meeting people report certain health effects which they “know” are caused by the exposure. At the same time, the communicator with years of technical experience “knows” that the claimed effect is not likely to be associated with the particular exposure. In a decade rife with alternative facts, it would be easy to be skeptical and dismissive of the former. However, certainty is no guarantee of correctness (Fischoff et al., 1977). Moreover, these represent fundamentally different ways of knowing, of incorporating memories, experience, and perceptions tempered with reason. Also, there is a distinction in law between “beyond a reasonable doubt” versus “more probable than not.” Scientists tend to have a high bar for accepting causal relationships. Epidemiology is intrinsically conservative (Gochfeld, 2003a) in making statements about X causing Y. Public audiences are much more tolerant and are more willing to accept causal connections than communicators are to assert them. In statistical terms, a risk manager's insists on 95% confidence for example, that trichloroethylene causes cancer, while the public is likely willing to accept a causality at 50% likelihood (Gochfeld, 2003a).

## 3 A PHYSICIAN'S ROLE IN RISK COMMUNICATION FOR CHRONIC OR CRISIS EVENTS

The present article invokes selected personal experiences to illustrate a variety of contexts, hazards, audiences, messages, time frames, channels, successes, and failures (Table 1). The risk communication literature often addresses a “crisis” or acute, emergent event where the “facts” are initially unknown or confusing as in the *Three Mile Island case*. On the other hand, environmental risk assessments often address events that evolve over time such as the *Maywood Thorium* example below. For environmental risk the “event” usually appears as a chemical, radiologic, or biologic hazard, but it may also be a *locally unwanted land use* (LULU; Greenberg, 1993), from a residential halfway house next door to a proposed nuclear plant. Among the cases discussed in this paper, *Roxbury & Fenimore Landfill* concerns a recent siting of a colossal LULU. I have little experience dealing with a not‐in‐my‐backyard (NIMBY) case. NIMBY is a crucial environmental justice issue in New Jersey and elsewhere. New Jersey's Environmental Justice Law (NJDOJ, 2020) requires permitting agencies to consider prior burdens on low‐income communities of color before permitting any noisy, unhealthful, or air polluting facilities to be sited within their borders. It recognizes that health concerns are often secondary to security, social, or aesthetic concerns.

**TABLE 1:** Risk communication case studies described in this article. Outcome reflects my final assessment. Arrow indicates whether my message was neutral(=), reassuring(↓), or activating(↑)

**Event and Audience(s)**	**Origin and duration of event**	**My entry**	**Duration of involvement**	**Directionality and outcome**	
*Three Mile Island* “meltdown” to NJDOH senior Public Health Officials. A true crisis lasting 5 days.	1979 (1 week) with a long “tail” of uncertain consequences	Day 2	1 week In person and intermittently for years	Neutral & confused at first then reassuring to avoid evacuation SUCCESSFUL	=↓
*Asbestos in schools* to school board officials and parents	1970s–1980s	2nd year	4 years In person meetings	Increase awareness of hazards and risks to promote safe removal practices MIXED	↑
*Asbestos removal worker training* to promote safety and safe behaviors	1978–1980 Pre‐AHERA	1st month	2 years 8 Classroom 2‐day courses	Increase attention to taking asbestos risks seriously UNKNOWN	↑
*Universal lead screening* in NJ children to pediatricians & family doctors	1995 NJ LAW	1st year	2 years about 12 Invited seminars	Increase awareness and compliance FAILED Revised message	↑
*Mercury Spill in Peru*. Town manager, mine company, local physicians, and community residents. A true crisis phase of about 8 weeks.	2000	4th week	1 month	Increase awareness of Hg vapor toxicity. And capacity building SUCCESSFUL BUT UNSATISFACTORY	↑
*Maywood thorium* (1950s until exposure ended 1985) DOE FUSRAP site. Meet with community residents	Historic (1940–1980s) but publicized ca. 1998	13 years. Discovered 1985 I entered 1998	1 year including lecture and planning intervention	Increase awareness and clarify benefits and limitations of screening and health promotion SUCCESSFUL	↑
*Chromium remediation in Jersey City* to community groups and local governments in Hudson County	1970s to about 2020	ca. 1990	20+ years 1990–2015	Increase awareness of cancer risk, explain rational for remediation and cleanup MIXED	↑
*Chromium in Garfield* 1 meeting with community residents for a lawsuit	1983–1993 groundwater discovered 2013	Months after public notice	1 night in person lecture format	Increase awareness of cancer risk. Increase participation in medical monitoring MIXED	↑
*Hydrogen Sulfide in Roxbury* Town leadership team, town residents, and NJDEP	*Hurricane Sandy* Oct 30 2012 to 2015	10th month	ca. 2 years 2013–2015 Meetings and email	Decrease for town and increase for State SUCCESSFUL	↑ ↓
*New school demolished* by outraged community. School board and parents	2000	1st year `	6 months	Decrease, “no exposure no risk.” FAILED	↓

Abbreviations: DOE, US Department of Energy; FUSRAP, Formerly Utilized Sites Remedial Action Program; NJDEP, New Jersey Department of Environmental Protection; NJDOH, New Jersey Department of Health.

In the examples that follow, I was sent or summoned, to discuss the various hazards and risks with one or more audiences. Audiences were not uniform or monolithic, but included people with and without exposure, with varying needs or uses for the information communicated and with various stakes in the exposure or outcome. Eventually I learned about the construct of mental models (Morgan et al., 2002), and I found this useful in thinking about how I could influence people's perceptions of a risk (Hampson et al., 1998). A person's mental models reflects their “understandings” of the hazard, exposure, and risk. These mental models can be eroded or strengthened by subsequent experiences or arguments, but often they become hardened and resistant to change. I realize that I, as the communicator, also have mental models which shape the way I communicate. Communicators may be risk averse (“the risks are worse than you think”) or risk tolerant (“there is no evidence of harm at this exposure level.”) A critic would call these biases to be exorcised, but they are unavoidable.

Physicians, particularly in surgical specialties, have to gain expertise in communicating risks such as probability that a procedure will be successful or that a cancer patient will be cured. This is not generally taught in medical school. As an environmental toxicologist and physician. I had the same challenge—‐no formal course in communicating probability. As a result, few of my examples even mention probabilities. Some of basic suggestions and questions a physician, toxicologist, or risk communicator should consider before embarking on a risk communication adventure are essential preparation for a risk communication opportunity. These are outlined below:

- Study the event: What is the hazard, who are the receptors, is there an exposure pathway, is there high, low or no risk. Review media coverage; look for EPA or Superfund or State environmental reports. Is there an Agency for Toxic Substances and Disease Research (ATSDR) report.
- Who are the audience(s): What are their concerns? What do they want to know? How much have they already heard? What is their role? Are they decision makers?
- Is there a basis for outrage: What is known? What is uncertain? What is unknown?
- Master whatever science or risk assessment information is available.
- Be prepared to answer questions and to say, “I don't know.” Practice questions you would ask in their place.
- For risky jobs: What has to be done? Is the work dangerous? Can it be done safely? Is there a safety assessment? Is there confidence in the safety assessment?
- What is the communication channel: a lecture with or without slides, roundtable meeting with officials, Zoom meetings, emails?
- Be clear about your role or intention: Are you correcting unwarranted fears, reassuring those with warranted fears, or arousing those complacent about risks?
- Think of a risk communication opportunity as a learning experience rather than a frightening chore.

## 4 CASE STUDIES ILLUSTRATING RISK COMMUNICATION CHALLENGES

The case studies I selected illustrate different kinds of hazards, audiences, health effects, communication channels, and roles that must be understood. These are summarized in Table 1. These examples are organized approximately chronologically because both the practice of risk communication and my experience and comfort evolved during the 40+ years covered by these examples. Risk communication in 2020 is very different in many respects from 1979. I end the narrative pre‐COVID, because COVID changed risk communication fundamentally as well as in practice, and that needs a different narrative.

Risk communication can be satisfying or frustrating. Just as there is often no clear beginning, community risk stories do not always have an ending. For example, crises such as *Three Mile Island* and the *Mercury Spill in Peru* have a long‐tail or aftermath even when the hazard is controlled. Apparent clusters of a disease, usually a specific cancer such as leukemia or cancer in general, are discovered by the public, and arouse concern in a community looking for answers. There is a science for studying space–time clustering (Jacquez et al., 1996; Wartenberg & Greenberg, 1993). The CDC (2013) provides guidelines for evaluating and communicating about putative cancer clusters. Often what appears to be a clusters of cases turns out to be a statistical coincidence, chance concurrence events, bound to happen somewhere some time. Understandably health and environmental agencies are reluctant to invest resources and energy at the first headlines reporting a “cluster.” There is often a delay between first reports and onset of investigation, during which the community's conviction that something serious is wrong may harden. As cases and public pressure mount, a study might be conducted. The truth about putative cancer clusters is that they usually fail to meet criteria for a space–time cluster, but this message, “it's a coincidence,” is not always welcome or believed. It is the numerator versus denominator conundrum. Anyone can count the number of cases, particularly with the aid of social media. “I can count ten cancer cases.” But epidemiologists emphasize the denominator. How many such cases would be expected there and then.

Sadder still, except for workplace cancer clusters, the relatively few events that constitute statistically validated clusters, are usually dead ends when further study fails to find the smoking gun, the cause. This leaves the communicator and community without a satisfactory answer. The Rutherford NJ, leukemia‐Hodgkin's disease cluster in the late 1970s, proved to be a statistically significant time–space clustering. The cluster, particularly the five leukemia cases in a single school, attracted national attention (O'Toole & Lescaze, 1978). The New Jersey Department of Health concluded that there was little likelihood (*p* < 0.001) that this was a chance coincidence. However, detailed case tracing, a formal case control study (Halperin et al., 1980) and environmental measurements of air, soil, water, buildings, using 1980 technology could not identify a cause (Burke et al., 1980). The final message from Trenton “we do not know” was unsatisfying to both parents, students, and health officials. In 2021 I asked Burke (pers. comm) and Halperin (pers. comm) about the possibility that today's environmental science might have uncovered a likely cause. The consensus was, “possibly but not likely.”

The counterpoint to Rutherford was the childhood leukemia cluster investigated at Toms River, New Jersey. A detailed health department investigation concluded that prenatal exposure to two environmental factors in the past were associated with increased risk of leukemia in female children. The sources were contaminated drinking water and industrial air pollution. Community frustration and scientific quest were documented in the Pulitzer Prize winning book *Toms River* (Fagin 2013).

Table 1 summarizes the case studies used in this article. The first two events in the table occurred while I was at the New Jersey State Department of Health, heading a new Environmental and Occupational Health Division. I left the NJDOH in late 1980 to join the newly formed Department of Environmental and Community Medicine, chaired by Bernard Goldstein MD at the Rutgers Medical School. Dr. Goldstein with Michael Gallo, PhD, developed the first Toxicology program in New Jersey. Goldstein led formation of the School of Public Health, and also formed the multicollege, multidisciplinary Environmental and Occupational Health Sciences Institute which provides a variety of environmental health services and expertise to New Jersey and beyond. EOHSI is now part of Rutgers University Biomedical and Health Sciences (RBHS).

In Table 1 “Directionality” refers to whether my role was to increase (↑) or decrease (↓) the audience perception of risk. In some cases my role was neutral (=) and in some cases dual (both directions). Only two events (*Three Mile Island* and *Mercury Spill in Peru)* represent crises (sudden onset, short term involvement). Outcome was “Successful” if the communication achieved the intended effect (i.e., no evacuation in NJ during TMI). “Failed” indicates that the communication objective was not achieved. These terms are elaborated under each case.

### 4.1 Three Mile Island nuclear meltdown (March 1979)

On March 28^th^ 1979, and over the ensuing five days, the Three Mile Island (TMI) Nuclear plant, near Harrisburg, PA, underwent a major cooling failure crisis with venting of radioactive gas and a partial meltdown. Conflicting information had led operators to shut down water flows when they should have been feeding water to cool the core. Valve failure coupled with human error contributed to the steadily worsening situation over several frightening days (Presidential Commission, 1979).

I had been at the New Jersey Department of Health, barely 4 months as head of Bureau of Occupational and Environmental Health. I was among several Department of Health people called to meet with the Commissioner. My role now was to keep Commissioner, Joanne Finley MD and senior management (four physicians and a senior journalist) up to date on the evolving crisis, the threat to New Jersey, and whether particular actions such as massive evacuation or shelter‐in‐place was required for New Jerseyans. The team had to make these decisions in the face of bad information and changing information. Radioactive gas had escaped on day 1 with no estimate of what or how much. TMI is 100 miles west of New Jersey or, depending on your mental model, “only” 100 miles west. On day 1, the utility had downplayed the crisis and gas release, claiming everything was under control. But it was not. It was a real crisis.

“Things are happening fast,” NJDEP Assistant Commissioner, Glenn Paulson told me early on day 2. “They [Pennsylvania] are considering evacuating thousands of residents.” “A meltdown is possible.” I read up quickly on “meltdown consequences.” Not good. And not so easy to research in preinternet days.

Day 2 saw conflicting and chaotic news reflecting the actual chaos on the ground. The utility's news source was unreliable. The Nuclear Regulatory Commission (NRC) representative arrived, but NRC, too released reports, which though authoritative sounding, were also mere guesses. Media flocked to TMI hungry for stories. The morning news emphasized that the radiation release had spread for miles (Sandman & Paden, 1979).

On day 3 a giant, potentially explosive hydrogen bubble was detected. News reports threatened a massive explosive release of radiation. The NRC recommended evacuation of at least a 10 mile, possibly a 20 mile radius, if the situation worsened (Presidential Commission, 1979). It did not define “worsen.” Governor Thornburgh was more worried about the potential risks associated with emergency evacuation, and rejected the NRC recommendation. By the end of day 5 the crisis seemed to have passed.

I had very little radiation training beyond the classroom, and felt my own limitations as I tried to sift the very sparse wheat from the media chaff. The utility had lost credibility on day 1 and NRC had yet to earn it on day 2. I explained or clarified radiation effects, reviewed the terminology that was being tossed about, such as “rads” and “rems,” and “curies” and we examined potential exposure pathways to New Jersey. My audience had two questions: “What do we say publicly”? and “When and where should we recommend or require evacuation?” There was no quantitative information on how much radiation had been released on different days. A hydrogen explosion was the major threat for a radioactive release on day 3. The main concern was that a cloud of radioactive gas could deliver a medically significant dose of iodine‐131 to New Jerseyans. Iodine‐131 is a very "hot" isotope with high specific activity. The term “radioiodine” that covers a variety of isotopes with very different properties, but it is the iodine‐131 with its 8‐day half life and its affinity for the thyroid gland, that is of immediate concern.

I led a discussion with the Commissioner and her staff, of the compound risk that (1) an explosion or meltdown would release radioactive gas, (2) that a gas cloud with a significant amount of radiation could reach, New Jersey, and that (3) this would result in significant exposure, dose, and risk if people were not told to evacuate or shelter in place. We had no numbers for any of these, but collectively we agreed to keep an eye on #1, the threat of explosion. We were reassured by the limited evacuation recommentations‐ from Pennsylvania. By day 2 Governor Thornburgh encouraged people close to the plant to leave. On day 3 he urged evacuation of pregnant women and infants living within 5 miles. He issued a stay indoors message for other people living within 10 miles (Presidential Commission, 1979). Eventually over 200,000 people evacuated, many from Harrisburg about 12 miles from Ground Zero.

This was the world's first large scale nuclear power plant accident. There was no “playbook” (Sandman & Paden, 1979). The Nuclear Regulatory Commission was not prepared. The TMI disaster met an unprepared public and a naïve but energized media grasping at occasional updates from the plant. There were no cell phones, no internet, and not even cable news channels (CCTA, 2021). Television offered pundits with a lot to say based on little verifiable information and no prior experience. It was a world of guesses. The official statement embodied in the Presidential Commission report (1979) is that the total release of radioactivity to the environment would have produced a negligible cancer risk that could not be discerned from background. This appears more as an assumption than a conclusion from data. This finding remains controversial today.

What I remember most about TMI was having to convey to Finley, the conflicting and rapidly changing information. The utility company eventually stopped talking, having lost credibility after circulating misinformation on the first 2 days (Sandman & Paden, 1979). The NRC acknowledged that this was an unprecedented event, and later investigations revealed that there was indeed no plan for such an event. Local government did not have a plan to trigger or manage an evacuation. This was uncharted territory for an unprepared industry and society. The detailed media analysis by Sandman and Paden (1979) later confirmed the suspicion that there was no reliable authoritative voice any time in the crucial week. After the crisis passed, the media undertook detailed analysis of the events, causality, and relation to the future of nuclear energy (e.g., Washington Post, 1979).

The near meltdown of the *Three Mile Island* nuclear plant was a very real environmental crisis that still elicits controversy over the “facts” 40 years later (Reinhart, 2019). It was really the first nuclear disaster at a commercial power plant. The nuclear industry would take years to recover, as dozens of new plant applications were put on hold (Presidential Commission, 1979). Also, there was a significant priming event—the release 10 days earlier of the *China Syndrome* movie with its high‐profile stars (Jane Fonda, Jack Lemmon, Michael Douglas) investigating an accident and cover‐up at a hypothetical nuclear plant. Conjunction of The *China Syndrome* plus TMI helped shape a generation's view of nuclear safety, in which nuclear industry experts could not be trusted (Southwick, 2019). TMI's industrial representative proved that point on day 1.

Shortly after arriving in Trenton, I had raised the idea of issuing potassium iodide (KI) tablets to people living around New Jersey's two nuclear plants. During the crisis it would have been helpful to have a KI supply available. I explained about I‐131, thyroid cancer risk, and I had pushed the blocking role of KI, which could keep I‐131 out of the thyroid. We had learned that KI was not a standard stock item, but could be special ordered. The Commissioner, however, had nixed the KI suggestion. “People might take the pill at the wrong time,” she worried. The Presidential Commission (1979) concluded that I‐131 release had been negligible, and the only health effects it acknowledged was “stress,” lots of it induced by bad information and alarmist media coverage. The report left an impression that the “media was the problem, not the plant, certainly not nuclear energy.” I found this unsatisfying at the time and it is contentious today.

In subsequent communications with the Commissioner, after the TMI crisis had passed, I explained the efforts being made to reconstruct radiation exposure and to initiate epidemiologic studies around TMI. I reported that Pennsylvania had initiated an exposure registry of people living close to TMI as a prospective cohort. I suggested that although this was an appropriate undertaking, it had limited sensitivity to detect an event‐related cancer even close to the plant. The initial assessment was that radiation release, particularly I‐131, was only slightly above background, projecting less than 1 additional cancer case (Presidential Commission, 1979). We congratulated ourselves for staying calm, monitoring the chaotic information, reassuring New Jerseyans, and not recommending evacuation. The absence of significant health effects remains the official understanding of the consequences of the TMI accident. The prospective registry study continued for 17 years and did not find elevated cancer incidence (WNA, 2020). As a footnote TMI ceased operation in 2019 (Sholtis, 2019). Nuclear power, once promoted as “too cheap to meter,” was becoming economically unsustainable. One outcome is that for more than a year before TMI, New Jersey had been planning to develop a statewide cancer registry. It is likely that TMI added impetus, and the registry was initiated in 1979.

### 4.2 Asbestos in schools

In 1978, about half my job at the Department of Health was involved with the hazards of asbestos‐insulating materials in public schools. Asbestos refers to a group of minerals that form microscopic fibers that can confer fire resistant and friction resistant properties to materials that have many applications, particularly in construction. At one time, construction codes required asbestos for fireproofing new buildings. Unfortunately, the microscopic fibers are also very dangerous when they get into the lung. They can cause lung disease (asbestosis), lung cancer, intestinal cancer, and mesothelioma. Asbestos is so potent, so dangerous, that about one‐third of asbestos workers die of one of the asbestos‐related diseases (Merlo et al, 2018; Selikoff et al., 1979), varying by industry and job type.

The post‐World War II building boom had included construction of many new schools. Economics had favored construction with flat roofs (rather than pitched) and asphalt rather than coal tar. Fire codes required the use of asbestos on construction steel and piping, and even favored asbestos ceilings. Flat, asphalt roofs eventually leaked so after a while schools with leaky roofs had water‐damaged shredding asbestos ceilings, exposing staff and students to the dangerous fibers. As asbestos epidemiology in the 1960s‐ and 1970s (Selikoff et al., 1979) reported the high disease potential, there was collective worry about what to do to protect school occupants, how to safely remove damaged asbestos, or manage intact asbestos in place. The costs could be a great burden to a school district. School districts had to make decisions about removing asbestos or managing it in place and EPA (1979) offered guidance which was updated (i.e. changed) periodically until the passage of the Federal *Asbestos Hazard Emergency Response Act* (AHERA, 1986), which imposed more specific and often costly requirements on school districts.

Schools planning expensive removal jobs had to apply to the State Department of Education for approval. The Commissioner of Health and I convinced Department of Education to require that schools hire trained asbestos removal workers (see *Training Asbestos Removal Workers* below), rather than endanger their own custodial staff or hire students. My job along with an industrial hygienist, required touring schools with their principals or managers to help them evaluate where asbestos was known or likely to occur, and determine whether the condition and accessibility of the asbestos rendered it an immediate hazard, or whether it could be managed in place. I would identify places where bulk samples were required for microscopic analysis to determine whether the substance was actually asbestos. I attended public meetings where I presented what became a well‐practiced lecture (no slides) on the nature of asbestos materials, its release from different construction materials, how people are exposed, what happens to asbestos fibers in the body, and how frequently asbestos contributed to various types of cancer. Intact solid or “cementitious” asbestos substances were managed in place, since removal risked exposing workers and spreading fibers. Water‐damaged, cottony “friable” asbestos had to be removed safely by trained workers. This was a risk communication challenge (Gochfeld, 1997). Apart from teachers and staff with full‐time exposure, schools are important workplaces for children for a dozen formative years, and making them safe has become an area of public health in itself.

My audiences were school boards, teachers, and parents. There was seldom outrage. In fact, usually there was not much interest in my risk message. "Why are you telling us this?" I was asked. Discovering asbestos in their school was a problem. Having someone from Trenton telling “You have to do something” would entail expenses. Districts were concerned about costs. This became an environmental justice issue as less affluent districts faced unexpected costs. Who would pay for asbestos management or removal, particularly after I explained the extra safety procedures required for safe removal. In the 1970s fear of asbestos and its microscopic fibers was not yet widespread. That changed drastically by 1980. Occupational exposures carried high risk, while household exposures were typically low. Schools as a workplace for teachers and students were intermediate and the risk level could only be extrapolated from higher level industrial exposures. At first, I used the statistic that one‐third of asbestos workers died from one of the asbestos‐related diseases. But no one identified teachers or even asbestos removal workers, with lifelong asbestos workers, so I could tell the audience was unimpressed by the statistic. I dropped it from the talk. Risk communication has to be adaptable. Over several years I spoke with about two dozen school audiences throughout the state. This activity continued after I left the Department of Health (late in 1980) and migrated to Rutgers Medical School to join Dr. Bernard Goldstein, in forming the Environmental and Occupational Health Sciences Institute (EOHSI).

When the *Asbestos Hazard Emergency Response Act* (AHERA) was passed in 1986, it required a formal management commitment by each school district, and use of trained “asbestos workers,” rather than relying on school custodians to remove asbestos. Many school districts did successfully remove or manage asbestos in their buildings. Many never did (NJEA, 2016). EPA has not made school asbestos a priority for inspections (EPA, 2018).

### 4.3 Communicating risks to asbestos removal workers

In the late 1970s, as asbestos in schools garnered headlines, the Department of Health persuaded the New Jersey Department of Education to require schools to hire trained asbestos workers for any removal jobs above a certain dollar amount (probably ca. $10,000). The Education Department agreed to use its budgetary authority, as long as there were trained workers to be found. I felt a “put up or shut up” responsibility. I teamed up with an industrial hygienist from the US EPA to develop a 2‐day course that included formal training in the methods for safe removal and disposal of asbestos, as well as details on exposure, protective equipment, and the health risks. The National Institute for Occupational Safety and Health (NIOSH) had circulated a very useful slide show on asbestos hazards in different occupations.

Our risk communication message was that asbestos is a potent carcinogen, which is why it was being removed, and it has to be handled carefully with appropriate protective equipment to keep the removal workers safe (Balmes, 2013). Even a brief exposure (weeks) is sufficient to cause mesothelioma decades later. If maintenance or removal of asbestos were necessary, the school had to contract with a trained workforce, familiar with the risks posed by asbestos and how to protect themselves and prevent wide spread of asbestos contamination of school buildings. Although construction workers, sheet metal workers, and others might have some experience removing asbestos, they usually had only a faint idea of the risks. Without special training they were at increased risk (Landrigan, 1991).

We used course material developed by EPA and NIOSH and supplemented this with original graphics. I taught half the course as basic risk communication in lecture format. The lectures covered the physical properties and pathogenicity of asbestos, exposure routes, fate in the lungs, and the pathology of asbestosis, lung cancer, and mesothelioma. It was a grim message, particularly the part about greater than one‐third of asbestos workers dying from some asbestos disease. No one walked out. We emphasized the importance of protective equipment particularly respiratory protection. This was complemented by the training half of the course which reinforced the importance of proper use of protective equipment, as well as proper removal techniques to limit the dissemination of airborne fibers. The practical included modules on air and bulk sampling, and even what duct tape and plastic sheeting to use to prevent spread through the school's ventilation system. We issued an attendance certificate and submitted a list of trained workers to the State Department of Education, which would allow schools to find these workers for removal jobs.

We ran these courses about quarterly with 40–60 workers over a 2‐year period (ca. 1980). This was not neutral communication. We wanted our trainees to be more concerned and take more precautions. We feared the trainees might become cavalier and careless around asbestos. So, our message aimed to raise concern, and undo complacency. “Take asbestos risk seriously as if your life depends on it.” When I left the NJDOH in 1980, there was enough demand that commercial trainers began to offer the courses, under approval by the Department of Health. This accelerated with the passage of AHERA (1986). The requirement for training certification for asbestos removal contractors and workers persists today (NJDOH, 2018).

Even with a trained workforce, the removal of asbestos from schools was a slow, cautious, and expensive proposition. In a 30‐year review of the AHERA program, the New Jersey Education Association (teachers) concluded that AHERA was well‐intentioned and comprehensive, but was largely ignored by school districts (NJEA, 2016), many of which used their custodial workers as asbestos removers. It is hard to assess the success of our worker training communication, except that it became a model for subsequent professional asbestos training programs approved by the State.

### 4.4 Universal blood lead testing of toddlers—Communicating with physicians

Childhood lead poisoning was a major public health crisis of the 1960s–1970s (Rosen, 1995). We knew that lead was bad for children especially, but since the average lead level in the population was 15 micrograms/deciliter of blood (μg/dl) we really had no clue of just how dangerous lead exposure was for children. At one time, 40 μg/dl was considered safe for children, and that unfortunately still is an allowable level for adult workers (OSHA, 2022). The lead story changed with the phasing out of lead from gasoline, eliminating the main contribution to environmental lead. Annest et al. (1983) published data showing that as the amount of lead used in gasoline declined through the late 1970s, the average blood levels declined as well from a mean of 15 μg/dl to 10 μg/dl. That decline continued through the next 30 years until the mean level is now below 2 μg/dl. But some children still had elevated blood leads that may interfere with their neurodevelopmental growth and cognitive development (Bellinger, 2008). Since the 1980s as lead levels have declined, the criteria for “elevated” level declined as well to 25 μg/dl, and 10 μg/dl (Rosen, 1995), then 5 μg/dl and now to 3.5 μg/dl in 2021(CDC, 2022). There is a consensus that there is no safe or natural level of lead in children's blood (Bellinger, 2008; Rosen, 1995).

In the 1990s, there was scant data on the distribution of blood lead levels in children. One of the aggressive recommendations from the CDC (1991) was for virtually universal blood lead testing of all toddlers. In 1995 New Jersey was one of a dozen states that mandated universal testing by law. Implementation was uneven (Goldman et al., 1998). Pediatricians balked at universal testing, in favor of targeted testing. A frequent comment was “My white middle class community doesn't have a lead problem.” Indeed, lead is an *environmental injustice* pollutant. It was and remains true that minority communities in old inner‐city housing are at higher risk of elevated lead levels than others (Gochfeld & Burger, 2011). However, "targeting" assumes that the doctor takes the time to evaluate risk to decide whether a family is “in” or “out” of the testing program. Only about half the NJ pediatricians and family physicians surveyed were testing a majority of their patients for lead (Goldman et al., 1998).

To promote the New Jersey's Universal Testing law, EOHSI was funded by the State of New Jersey to provide educational seminars to physician groups and medical societies that are always looking for continuing education speakers. I gave about a dozen seminars over a 2‐year period. Many doctors do learn about childhood lead poisoning in medical school, because many teaching hospitals are located in urban areas where lead poisoning is or was common. However, they graduate with the reinforced mental model that lead exposure is a disease of urban minority children. The theme of our seminar was to communicate the evidence regarding lead effects at levels once considered “normal,” and persuade primary care doctors and pediatricians to conduct universal testing for their patients. New Jersey law required two tests before age 3. The State could not document the prevalence of elevated levels without universal testing. The State and the CDC wanted the data on lead levels in upscale towns as well as inner cities.

My presentation was greeted politely (usually) but with skepticism. Targeted testing made more sense to an audience whose mental model was “Black children in inner cities eat lead paint.” However, targeting requires performing a risk assessment, beyond looking at skin color. The preventive measures often involve removal of lead contamination or even removal of children from contaminated housing, which involved legal actions beyond the scope of primary care practice. The NJDOH and local health departments had that responsibility. Older pediatricians, remembering when lead levels were much higher, were the least willing to consider universal testing (NJDOH, 2019) when average levels were already below 5 μg/dl. Most of our audiences were predominantly white, but black physicians also questioned the utility of universal testing.

My intended communication was unsuccessful, and after a few sessions I revised the message, because even targeted testing, a fallback recommended by CDC (1991), was not reaching deeply enough into the pediatric practices. My new message was more congruent with the audiences and their mental models. I emphasized the growing evidence that lead levels once thought normal were now clearly harmful (Rosen, 1995). To avoid a growing nihilism, the talk emphasized the successful public health steps for protecting children with elevated lead and their siblings, clarified the availability of health department intervention, and documented the long‐term decline in lead levels. This was clearly an environmental justice issue, and the physicians were all for protecting children. Blood lead testing is widely conducted in New Jersey, and the United States today, but is hardly universal. Between about 85–90% of 3‐year‐olds have had one blood test. About 2.5% have elevated blood leads above 5 μg/dl (CDC, 2022; NJDOH, 2019). Overall, this communication had mixed outcome, since the original intent failed, but physician participation in testing increased.

### 4.5 Mercury contamination in an Indigenous Peruvian village (June–July 2000)

Peru is among the top gold‐mining nations (United Nations, 2021), and *Minera Yanacocha*, perched on the altiplano at > 4000 m altitude, above Cajamarca city, is one of the largest of the country's gold mines. A Denver company, Newmont, is the main owner of the mine. In 2000, in addition to gold, the mine extracted mercury which was sold commercially, mainly for use by small‐scale artisanal gold miners in the lowlands (CAO, 2000), where it becomes a serious health threat for the miners, their families, and communities (Yard et al., 2012). On June 2, 2000 a truck carrying containers of mercury from the mine, careened down the winding mountain road. Its cargo of canisters was not properly secured. The load shifted and at least one of the mercury canisters began to leak, spilling an estimated 150 kg of elemental mercury liquid along about 40 km of the roadside, including in the largely indigenous villages of Santa Elena, Choropampa, and Magdalena (CAO, 2000; Moeys, 2020). In many cultures, including here in Peru, elemental mercury, the silvery liquid, is imbued with valuable, magical, or medicinal properties. Discovering the spill, villagers rushed out to scoop droplets of mercury into containers to take home. Even a few cc of mercury in an open container can saturate the air in a small bedroom with a toxic level of mercury vapor. This is worse in a hot climate, even without heating the liquid (Moeys, 2020). This collecting of the mercury emerged as a substantial environmental justice issue.

It was 2 days before the villagers received warnings from the mine company, that the mercury was poisonous. People were urged and later incentivized to return the mercury, a message reiterated over the next months as dozens of people got sick (CAO, 2000). Choropampa had a population about 3500 people in 2000. There is no estimate of how many people collected the roadside mercury. The best estimate, about 1000 people exposed, and 200–300 sickened, seems to have become the official figure (UNEP, 2013). The International Finance Corporation had a stake in *Yanacocha* and investigated the spill response (Taylor, 2000) as well as appointing a compliance/advisor ombudsman (CAO) to work with the community (CAO, 2000).

At the turn of the century (ca. 2000) Rutgers University's EOHSI was engaged in global environmental health on several fronts (Gochfeld & Goldstein, 1999). Newmont Mining, one of the largest gold mining companies in the world asked EOHSI to help in Peru with the exposure and toxicity assessment. What were the actual risks from elemental mercury and how bad were they? As presented to us, this was a straightforward mercury spill/mercury exposure case requiring evaluation and risk communication. It turned out to involve complex beliefs and practices of the indigenous communities, creating prolonged household mercury exposure. We were aware of these unusual practices regarding mercury in some U.S. communities, and EOHSI had a history of evaluating ethnic and cultural influences on mercury exposure (e.g., Burger et al., 1991). We were fortunately prepared for some of the surprises we encountered in Peru.

Dr. Paul Lioy and I visited Choropampa, arriving in early July, a month after the spill. From Cajamarca city we drove over a winding road through spectacular mountain scenery. Before reaching the village we saw road workers shoveling mercury contaminated soil into bags as part of a decontamination and retrieval effort.

**FIGURE 1:**
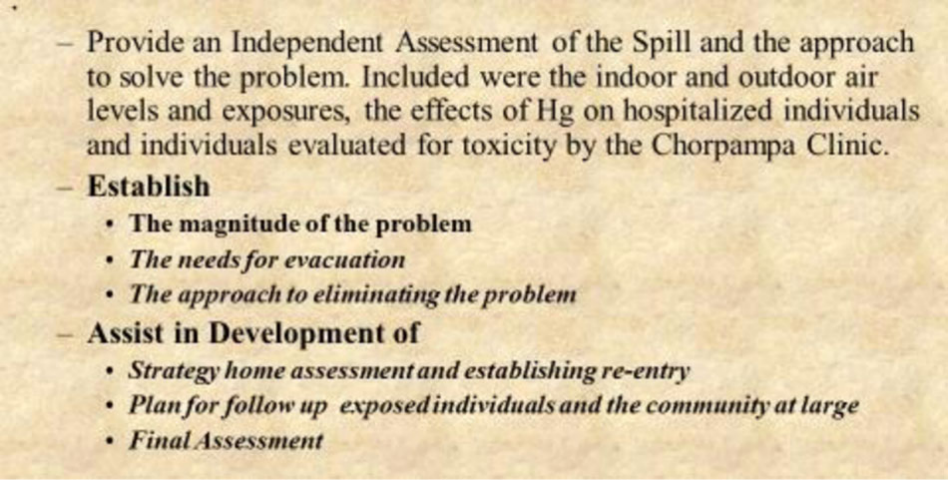
The charge for Gochfeld and Lioy at Choropampa, Peru, July 8, 2000. We were responsible for “an independent assessment,” but also for help with the details of a reoccupancy protocol. “The approach to eliminating the problem” turned out to be much too broad and long term

We had received a company briefing and an assignment, which seemed overly broad and very optimistic (Figure 1). In town we were met by a contingent of Newmont's technical people including, environmental contractors, and by a public health official from Cajamarca, local doctors, an analytic chemist, and the town mayor. Most of them had been on site for weeks, and some knew the victims personally or had even collected mercury themselves. This would be our audience over the next few days, not exactly a team—its individual roles and compositions changing. The briefing provided us with extensive detail, a map showing contaminated houses including a street‐by‐street review of where mercury had spilled in town. Eventually the company opted to tear up and repave the contaminated roads. Our far‐reaching assignment gradually came into focus. We were here as consultants and risk communicators on issues of toxicology, medical diagnosis, and also on exposure assessment, analysis, and cleanup. But we were also seen to be representing the interests of “the mine,” which left a cloud of distrust over our messages.

We conversed mainly through an interpreter. My Spanish was pretty limited to “el vapor del mercurio es muy venenoso.” I had many opportunities to use that phrase. A “communication” offers the opportunity to learn as well as teach. Over the preceding month since the spill many villagers had developed symptoms attributed to mercury poisoning. In the first month, nine cases of contact dermatitis attributed to mercury, were physician confirmed. This is not a typical manifestation of mercury exposure. In the month before we arrived “a steady stream of people sought medical care for symptoms of mercury intoxication” (CAO, 2000). Many had been hospitalized at the regional hospital in Cajamarca. Those unable to return because their homes were on the contaminated list, had been housed in a hostel in Cajamarca. One woman had gotten acutely ill, inhaling hot mercury vapor while waiting in vain for the gold to reveal itself. Despite or perhaps because of aggressive chelation with penicillamine, she had become comatose, and had been evacuated to Lima. She never recovered (Moeys, 2020).

The biggest communication challenge was to bring the team together on the issue of mercury vapor toxicity. We were told that for the first week or so, the authorities had not been aware that elemental mercury releases a toxic vapor that sickens by inhalation. Apparently, this was true of some of the mine representatives, not just the community. The main prevention message had been to avoid skin exposure. In the initial warnings, people were told not to touch the mercury, but nothing about breathing vapors. Many in the town had handled the mercury while collecting it. Even a month later, some audience members still had no clear understanding or belief about how elemental mercury releases an invisible, odorless toxic vapor indoors. On hot days, vapor generation would increase. My initial explanation in both English and Spanish was not entirely successful. I could sense the mayor's disbelief when I explained that mercury liquid will evaporate releasing a gas that is invisible and odorless, and highly toxic.

Partly there is something quite incredible about how mercury toxicity works and partly, I feared our credibility was limited due to the association with the mine. We explained that mercury vapor, invisible and odorless, can accumulate in enclosed spaces indoors and build up to toxic and even lethal levels. Dermal absorption, I explained to obvious disbelief, is negligible, and mercury is not absorbed from the intestinal tract. I resisted the temptation to demonstrate. As company representatives, anything we said was suspect to some people.

While Lioy was reviewing the household sampling protocol and exposure assessment, I asked to see some of the individuals with “mercury poisoning.” I was told they were still in Cajamarca waiting to come home. I was told about a data base of cases, but it was not available initially. We were asked to focus on what mercury level in a house was low enough for re‐occupation or conversely what was too high to allow people to return. The team developed a classification of mercury air concentrations (Table 2), that we agreed with. However, we felt the need for a standardized sampling protocol to generate reliable data on whether a house was indeed safe for reoccupancy. There was still work to be done in decontaminating houses in Chorapampa.

**TABLE 2:** Indoor air standards for reentry and remediation of homes. Developed by an environmental health team in Choropampa, Peru representing the town, the company, and EOHSI

Levels	Designation	Comments
LEVEL 1 > 0.003 ppm Hg	Unacceptable for habitation without remediation	Initially 39 houses Level 1 Maximum concentration: Four values exceeded 0.02 ppm
LEVEL 2 0.001 TO 0.003 ppm Hg	Short term habitation (weeks) allowed but requires remediation	19 houses
LEVEL 3 0.0004 PPM to 0.001 ppm	Habitation allowed but requires further assessment, hot spot identification and remediation	19 houses
LEVEL 4 < 0.0004 ppm	Clean! No further assessment or remediation required	Most of the houses that allowed sampling. No accurate count

The distrust and sometimes hostility were obvious in the interactions of company, town, and health representatives. The company technicians were suspect and so were we. The skepticism about our mercury vapor story reflected this lack of trust. The public health technician with a Jerome mercury meter and the mine company technician with a Lumex, were both measuring mercury vapor concentrations in houses, but they were not sharing results or even calibrations. Lioy encouraged them to do some paired readings together and explained the limitations of the Jerome detection level. This was the device used by the public health technician. Lioy recommended the use of a Lumex with its much lower detection level. Fortunately, the company provided an additional Lumex portable analyzer for the health technician. This generosity enhanced the company's image and greatly facilitated the search for mercury hots spots in houses, prior to classifying them. Now the two men formed a team of sorts with a common mission of measuring mercury levels in homes, determining which could be reoccupied, as well as trying to locate stashes of mercury.

Villagers who wanted to keep their hoarded mercury, locked their doors so their homes could not be tested (CAO, 2000). Their owners may even have left town. More troublesome was the conflicting information that persisted about vapor. Even after a week, we heard from two different people that “this type of mercury is not dangerous” (CAO, 2000, p. 18). They did not believe in the invisible vapor. At one of the daily meetings, we demonstrated how liquid mercury releases an invisible vapor. We showed how the Jerome meter needle would move when the wand was held near or over open containers of mercury. We covered the containers and repeated the test. The needle did not move. We ran the test multiple times with multiple containers, and gave the wand to people in the audience to repeat the demonstration. Not everyone was convinced with our explanation of the needle phenomenon, assuming it was some magic trick. However, one woman reported feeling cheated, because her mercury had disappeared after heating it over a stove, without revealing any gold.

As part of the standardized protocol on reoccupation, we needed reliable data. In order to classify individual homes according to Table 2. We developed a systematic sampling approach covering entry ways, corners, sleeping areas, and cooking areas. We explained to the team and some of the homeowners that after 4 weeks, the vapor could have seeped into the soil of bare floors and into coarse construction material of the building which had become, in effect, a secondary source. Mercury vapor is heavier than air accounting for its presence in floors or close to floors. Even when all visible mercury had been removed, high mercury readings were obtained in some homes. Heating the mercury, apparently in hopes that it contained gold, had increased its vaporization and dispersion. We were late arrivals in a decades old history of villagers distrusting the mine as well as coming a month late to the mercury spill story.

One question: Would clothing that adsorbed mercury vapor have to be destroyed? We called on our experience with mercury spills in an urban residential building in New Jersey. We had recommended a protocol of putting clothing in a plastic bag for 24 h and then measuring the headspace mercury in the sealed bag, as an approach for determining what could be salvaged or should be discarded. Unfortunately, large plastic bags were scarce in Choropampa and had to be brought from Cajamarca. If the mercury concentration in the bag exceeded 10 μg/m^3^ we explained, we would consider the clothing contaminated. This approach was later incorporated into ATSDR guidance for mercury spills (ATSDR, 2012; Gochfeld, 2003b). About one‐third of clothing samples exceeded this level. Although we recommended disposal as we did in New Jersey, most people chose to air out their contaminated clothing, trusting to days in the mountain breezes and sunshine. Choropampa's environment is nothing like New Jersey. We should have recommended airing out in the first place, another learning experience.

After retrieval from roadside soil, and a buyback program from residents, half the lost mercury remained unaccounted for (CAO, 2000, p. 22). The company assumed that much of this was presumably hidden by townspeople, although very likely much had simply dissipated in the environment. We were told that many townspeople still kept the mercury at home, and we suggested that without endorsing such behavior they should be warned to keep it in sealed containers. We learned that not every household had access to a sealed container.

I was particularly interested in biomonitoring, and was shown a summary of the results on 501 Choropampa urine samples tested by the Peruvian National Toxicology Laboratory. The results presented as PPB are equivalent to micrograms per liter. Note that 42 samples had mercury levels > 100 ppb; 299 in the range of 50–100 ppb; and 160 were < 50 ppb. Reference ranges for urine mercury vary among laboratories and references, but currently a level above 25 μg/L would be considered elevated. The high levels were consistent with the number of people reported to be sick. We asked if it were possible to link the mercury levels to individuals and households, to improve their utility and were disappointed that these data were not available.

When the household testing was completed, most of the houses we had access to in Choropampa had very low mercury levels, but there were 39 Level 1 houses, 19 level 2 houses, and 19 level 3 houses. By the time of our departure in late July, road cleanup and soil analysis was well underway, as was the decontamination of homes, air sampling, and classification of houses. As homes in Choropampa were decontaminated and mercury levels met the criteria in Table 2, people returned home from Cajamarca. Months later, we learned from Newmont, that two of the level 1 houses were still not habitable after multiple assessments and cleanings.

I did not have much opportunity to interview symptomatic people. A review of the reported acute symptoms included breathing problems (from heating mercury) sounded like mercury toxicity. The rash (identified as contact dermatitis), headaches, and tremors as well as a variety of other symptoms may have been due to mercury. Those same symptoms occurring years later were most likely unrelated to mercury (Arana‐Zegarra, 2009). In summary, we had ample opportunity to communicate with the town leaders, local physicians, and the company's staff, as well as some of the residents about the risks from mercury vapor. We hopefully convinced most of the skeptics that elemental mercury vapor is harmful. The visit was unsatisfying in several respects. We did not have the opportunity to link mercury levels in homes before cleanup with mercury levels in urine or with symptoms. We did not have the opportunity to distinguish people who merely stored mercury in their homes from those who heated it over a stove to find gold. I believe that most of the hospitalized patients would have been in the latter group. We were useful to the town in designing and validating the reoccupation strategy including standardizing the sampling. However, we felt that our linkage to the company undermined our effectiveness and may have tarnished our reputation. The most satisfying part was meeting with two young physicians who were a rich source of information about child health in the mountain villages. They were anxious to learn about mercury, its properties, and toxicity. I showed them some neurobehavioral tests I had brought with me, and demonstrated a grooved pegboard test with an enthusiastic child. I left the tests with instructions, for the doctors to use.

The ombudsman function continued for several years (CAO, 2006). There followed a long unhappy epilogue to the mercury spill, with many subsequent illnesses blamed on the spill, some plausible, others not (Moeys, 2020). The town's relationships with the Mining Company has been very stormy, with ongoing and new illnesses ascribed to the 2000 spill, and denied by the company (Moeys, 2020).

### 4.6 Thorium exposure in Maywood, NJ 1998–2015: A Department of Energy FUSRAP site

Disclaimer—I received an honorarium from a law firm for a talk to a New Jersey community audience, exposed to thorium dust. I described the properties of thorium and the risks from exposure to radioactivity in general and thorium in particular. I described the potential health effects including lung cancer and discussed the benefits and limitations of new lung cancer screening approaches. I explained how a medical monitoring program might benefit them. At the time of the lecture, I had no role in conducting the hands‐on program.

The former Maywood Chemical site in Maywood and Lodi (Bergen County, NJ) includes both a Superfund site and a DOE *Formerly Utilized Site Remedial Action Program* (FUSRAP) site (DOE, 1992). Cleanup is ongoing in 2022. One of the company's products was thorium, extracted from ore. Until the 1950s the Company supplied thorium‐232 to the Atomic Energy Commission. Thorium‐232 bombarded with neutrons produced the fissile isotope, uranium‐233, used experimentally in nuclear weapons tests (Friends of the Earth, 2015). The extraction and refining of thorium generated large quantities of radioactive alpha‐emitting dust and slag.

The history of the Maywood Chemical Site is iconic of industrial chemical production and toxic waste in the 20th century. Like the chromium companies mentioned below, the residual thorium waste was distributed around the neighborhood as fill for the construction of residential communities. The contaminated area included the former plant site, surrounding residential communities, and an 11 acre (4.5 Ha) radioactive waste site operated by DOE (DOE FUSRAP, 2022; EPA, 2022a). Several hundred private residential properties had some radiologic contamination. The extensive thorium contamination was “discovered” by DOE and EPA ca. 1985, and the community was notified. Therefore, many exposure activities including gardening and playing on dump piles, were believed to have ceased as of 1985. ATSDR (1990) concluded “that humans had probably been exposed to hazardous substances at this site at concentrations that may result in adverse health effects.” By the time lawyers contacted me (1997), about 100 private residential properties had been remediated. In most cases, the owners had had no idea that they were buying or living on toxic waste sites. In designing the monitoring program, we operated on the assumption that there was no significant radiologic exposure after the notification and cleanup of the 1985 period.

Lawyers organized a class action suit on behalf of the residents which included payment for cleanup and damages, and a medical monitoring component. I had previously designed medical monitoring for other legal settlements, and I was contracted to design the Maywood Medical Monitoring Program (MMMP). It was reasonable to assume that once informed of the hazard in 1985, people would have taken steps to minimize their exposure to thorium dust. We assumed that any cancer attributable to the thorium exposure would be manifest within 30 years after exposure ended in 1985. Hence, we explained, the MMMP would run from 2000 to 2015, encompassing the 30‐year latency for any thorium‐related cancer. Thorium is an alpha emitter and decays to radium and radon, therefore, inhalation of thorium dust as well as inhalation of radon gas, increase risk of lung cancer (ATSDR, 1990), hence lung cancer was a chief concern.

The rationale for the MMMP was the possibility of discovering a site‐related cancer at an early stage while it was still curable. Also, a comprehensive medical monitoring program provides opportunities for other health interventions or preventions which would directly benefit the class members, for example, regarding cardiovascular disease. This, in principle would offset their historical risk, even if they did not incur a thorium‐related cancer (Gochfeld et al., 2009).

Between 300 and 400 people who lived on the contaminated properties, including many who remembered playing on the waste piles as children, were considered eligible for the MMMP. Letters were sent to current addresses announcing two public meetings. About 250 people attended the two meetings at which I conducted a pretty traditional risk communication in lecture format. I reviewed the chemicals which had been found on the residential properties focusing on the radioactive thorium, radium, and radon. I reviewed radioactive risks and cancer, focusing on the inhalation of thorium‐contaminated dust and why lung cancer was the main risk, particularly for smokers. By that time (1998) the anti‐smoking efforts had been successful in reducing the adult smoker rate in New Jersey from more than 30% in the 1960s to less than 20% (RWJF, 2009) and to 13.5% in 2018 (NJDOH, 2022). Still the synergy between alpha radiation and smoking was a high‐risk factor for lung cancer in the Maywood cohort. Synergy is such an important concept to a toxicologist, but is not familiar to the public (Hampson et al., 1998). I also explained the advantages and limitations of the medical monitoring program. I diagramed the concepts of sensitivity and specificity, and predictive value. The audience understood the importance of finding something bad if it is there and also the concept of false alarms being bad. But it is not intuitive that you cannot maximize sensitivity and specificity at the same time. It was a challenge to describe the science and risk information. How successful was the risk communication: 300+ eligible, 250 attendees, 114 signed up, 71 showed up. So, it was moderately successful in encouraging participation in the screening program.

Although the original plan was to have the MMMP implemented by a medical group in or near Maywood, the participants expressed more confidence in having the MMMP conducted by EOHSI, under the supervision of Iris Udasin MD. She took on the responsibility for the patient visits and also for the periodic reassessment of the program, until the agreed upon closure date in 2015, 30 years after the last exposure.

In the 1998–2000 timeframe there was a widespread nihilism in medicine about lung cancer screening, despite widespread agreement that early detection improved survival. However, recent studies had found significant benefit using spiral CT lung scans for early detection of cancer (Henschke et al., 1999). A novel addition to the MMMP was annual low dose CT scans. CT scans carry their own radiation risk but the possibility of detecting and curing cancer in this at‐risk group warranted the CT. This was innovative at the time and required special discussion of risks and benefits. We would revisit and refine this component throughout the 15‐year course of the program. Ironically, when we concluded after a decade of negative CTs for most participants, that annual CT was no longer warranted (Bach et al., 2012), it was challenging to communicate to participants that the radiation risk from CT might now be comparable to their former thorium risk. Individuals balked at being told they would no longer get their annual CT.

The legal settlement provided for the responsible party to fund the monitoring program through 2015(30 years after the 1985 notification). Seventy‐one people began the monitoring program. Each year we prepared an annual report communicating to the participants any relevant findings (one lung cancer, six breast cancers found by the program), and any new information regarding radiation risk. The program was dynamic, incorporating new information regarding general health maintenance (cardiovascular, obesity, Mediterranean diet). Reinforcement of previous information was also included in the annual risk communication. The very few smokers received additional communications urging cessation and providing guidance.

In retrospect, my risk presentation was not alarmist, but was directional, encouraging people to participate in the MMMP without scaring them. Many people might have been in contact with thorium dust, without inhaling it. My emphasis on the smoking synergy, may have reassured the many nonsmokers that they were not at risk. Although I emphasized lung cancer as the special risk, I was cautious in not overplaying the potential benefits of CT scans, still not well documented in 2000. The MMMP did detect cancers that were not previously recognized, but at first there were many false positive readings of the CT scans that required repeated scans in 3–6 months. Eventually as radiologists gained experience the repeats diminished (Rampinelli et al., 2012). The emphasis on collateral health benefit, may not be persuasive to everyone. I did not record the lectures, unfortunately, but I did wonder whether if I had been in the audience, I would have signed up or not.

### 4.7 Chromium remediation in Jersey City, NJ: 1990 to 2015

The widespread chromium contamination of Hudson County, NJ, involved various EOHSI faculty for a quarter century, including many exposure and biomonitoring research components and periodic participation in public meetings with various affected communities (Stern et al, 2013). Communicating the risks of toxic chemicals is sometimes straight forward. More is bad. Some familiar metals: iron, magnesium, zinc, and copper are essential elements. Deficiency states are bad, and too much is not good. However, chromium is a particularly confusing element and as such its story is challenging to communicate. Chromium exists mainly in two ionic forms, the three plus charged state known as “chrome 3” or “trivalent” (Cr‐III) and the six plus charged state known as “hexavalent chromium” or simply “hex” (Cr‐VI). Trivalent chromium is considered an essential trace element, playing a role in carbohydrate metabolism. Hexavalent chromium is recognized as toxic, causing allergic skin reactions and also cancer. Inhalation of hex has caused lung cancer among workers in chrome plating factories.

From 1905 to 1976 three of the United States’ largest chromium refining factories were in Jersey City and Kearny, in Hudson County, NJ. Huge quantities of chromite ore had been processed there for industrial applications (Burke et al., 1991). After usable chromium was extracted, the resulting slag still contained hexavalent chromium, often at concentrations exceeding 1% or 10,000 ppm. The slag was disposed of by offering it free to communities anxious to expand by filling in wetlands. The fill got the reputation of killing any rats that ventured onto it, probably due to the residual chromium. There were over 160 chromate contaminated sites in Hudson County (NJDEP, 2010), and many were residential communities that would be undergoing remediation (NJIDEP, 2020). A Chromium Cleanup Partnership assumed responsibility for cleanups of certain sites, funded by one of the responsible companies, PPG (Chromium Cleanup Partnership, 2014).

My EOHSI colleagues and I met with the residents of several communities to talk about the unique features of chromium and explain the health risks attributable to hexavalent chromium. As soon as “carcinogen” or “cancer” was mentioned, they became interested in how to avoid exposure and how to get rid of it. They were not interested in whether the probability of getting cancer, the “risk,” was infinitesimal or just small. We did not spend time on probabilities. Nonetheless, some people balked at cooperating with the cleanup, fearing that they might lose their homes. Although we were explaining risks, the risks were actually relatively low and no fancy graphics would convince reluctant people to cooperate. We had better luck when we recruited people for a study that involved chromium analysis of dust in their homes (Black et al., 2015).

We also had multiple meetings with public officials of various towns within Hudson County. Some were outraged that the chromate companies had left behind so much toxic waste, and others felt that the companies had enriched the local economy for decades, providing jobs. Now the jobs would belong to the remediation workers. After remediation, homes near the cleaned‐up sites had significantly lower chromium in dust than prior to remediation (Black et al., 2015).

Of the almost 200 chromium waste sites eventually identified by NJDEP, some were parks, where removal of contaminated soil was easy. Others were covered by residential or commercial buildings. Over a 20‐year period my colleagues, Paul Lioy and Kathy Black, and I spoke at public meetings, conducted exposure studies, and became involved in legal issues over cleanup responsibilities. Early on (ca. 1990), we had to explain the analytic complexities of distinguishing Cr‐III from Cr‐VI. It was not straight forward. Depending on how samples were collected and processed, trivalent chromium might be oxidized to hexavalent, or hex might be reduced to trivalent. The same soil sample might be reported differently by two otherwise reliable, reputable laboratories. The need for “cleanup” or removal of chromium‐contaminated soil and the safety of the workers performing such tasks, hinged on correct analysis. This was hard to communicate to people unfamiliar with analytic chemistry. Indeed, it was hard sometimes to convince analytic chemists that they were not analyzing what they thought.

Subsequent meetings focused on cancer risk. Several groups performed risk assessments using different assumptions and their different approaches resulted in very different outcomes. By human health risk assessments, leaving 1% chromium behind in residential soil, resulted in cancer risks that were unacceptable. The NJDOH studied over 3000 lung cancer cases in Jersey City, finding a 10% higher rate among people living close to versus far from chromium contaminated sites (NJDOH, 2008).

A memorable public symposium was held at EOHSI at which competing risk assessments were presented, with conflicting conclusions regarding cleanup. I had the privilege or challenge of summarizing and explaining the results to the audience, to the media, and ultimately to the NJDEP to encourage further research. With a colleague, toxicologist Charlotte Witmer, I set out a series of unanswered questions, requiring new research (Gochfeld & Witmer, 1991). It was a complex message since there were many loose ends requiring further study. The primary audience was the NJDEP, but others included risk assessors and toxicologists, some of them hired by the Principle Responsible Parties (PRPs), the companies responsible for the pollution and financially liable for the cleanup. We identified research needs related to sampling, analysis, exposure, sources, fate and transport, bioavailability, toxicokinetics and toxicity, biomonitoring, epidemiology, and risk assessment. In other words, almost every area of environmental health required additional research relevant to managing chromium.

As giant remediation plans got underway, neighbors of contaminated sites were reasonably worried about whether the digging and trucking activities generated more dust in their community which they were then tracking into their homes. People were worried whether there was “hex” in their living rooms and bedrooms, perfectly reasonable concerns dealing with a carcinogen or any toxic substance. People kept asking and needed some answers. We designed a study to answer the question about the prevalence of hex in household dust. By doing intensive sampling of dust from homes in various communities where chromium was known to be present and in a reference community with no history of chromium industries we found little difference, until remediation began (Stern et al., 2013). We also offered to test people to measure chromium levels in their urine. After several years and studies, we were able to communicate reassurance to most people.

Generally, there were low levels of chromium dust in homes, even close to chromium contaminated sites. Most of the dust samples yielded only trivalent chromium. A few homes did have hexavalent chromium dust, some probably from remediation. In response the DEP tightened the controls on cleaning soil‐laden trucks as they left a remediation site to avoid contaminating roads and roadsides. We also traced hex to specific substances like paint and stain pigments used on some furniture that was independent of the soil contamination in the community.

Hanging over chromium remediation like a dark cloud was a legal case in which the responsible companies that had profited from chromium processing and from externalizing the costs of waste disposal, refused to pay their assessed share of the cleanup costs. In the end the State and Courts determined that the PRPs retained the liability for cleaning up the waste sites, digging deep into the soil where chromium had leached, reaching the water table (e.g., Honeywell International, 2010). By 2015 most of the large remediation jobs were completed and we were able to reassure many people that “hex” levels in homes were low (Black et al., 2015).

### 4.8 Chromium in Garfield, NJ – 2013

Disclaimer—I received an honorarium for a talk in Garfield that described the risks from having chromium in their basements, the potential health effects, and encouraged people to join a class action where appropriate. I had no further involvement with the case.

Chromium cleanup in Hudson County was prominently in the news in 2013 (e.g., McDonald, 2013), which probably primed the Garfield community (Bergen County) to pay attention to its own chromium contamination. The hexavalent chromium in Garfield was the same substance as in Hudson County, but the responsible party, a working electroplating company, was still functioning in 2013. The source in Garfield was a spill of thousands of gallons of chromium process waste water that had occurred in 1983, had been investigated in 1993, but was not brought to public attention until 2011 when the site was placed on the National Priorities List (EPA, 2021, 2022b), the Superfund List. By that time the chromium from the spill had migrated with groundwater and seeped through soil. The chemical properties of hex allowed it to seep through concrete walls into basements. A class action legal case was initiated on behalf of the families whose basements, and in some cases drinking water had been contaminated. The lawsuit included funding for biological monitoring of individual chromium exposure. I was invited to make a presentation to the community, particularly people close to the contaminated facility, to discuss health risks. There was remarkably little media coverage, but fortunately there was good background information on NJDEP's Superfund website (updated EPA, 2021).

I participated in one crowded public meeting in Garfield where I was asked to explain the source of hex and how it had moved with ground water into basements and why it was a problem. The hex on basement walls formed a fine dust which could be dislodged, become airborne, and inhaled. I explained why exposure to hex poses a health risk. I suggested that people who were fearful or who had lived in one of the dozen most contaminated homes, or had symptoms they thought might be related to hex, should sign onto the class action lawsuit and request a clinical evaluation. The attendees explained their experiences with contamination, and their frustrations with the company and the NJDEP. There was more of a sense of puzzlement over what to do next, rather than outrage, at the long‐delayed information not released until 2011.

Most people in town did not know if they had been exposed to chromium (Doherty Lyons et al., 2019). The several dozen people who attended the lecture were not a random group of citizens, but were interested enough to devote an evening to the topic. I discussed the cancer risk in general terms, without the benefit of house‐specific exposure information. Some of the audience had already had their water tested for chromium and were planning to install treatment systems. There is evidence that ingestion of hex causes cancer (Welling et al., 2015), but there is more concern about people breathing dust left behind as the water that had entered their basements evaporated. I did not try to address the probabilities of getting cancer from past exposure, or from ongoing exposure, except in qualitative terms. “Cancer” was enough to convince most of the audience to sign up for the class action, and many of them would subsequently participate in biomonitoring by having toenail clippings analyzed for chromium. The EPA eventually completed a cleanup of basements. The former industrial site was demolished and capped. However, treatment of the half‐mile long groundwater plume was long delayed until 2021 when funding became available under the Infrastructure bill (EPA, 2022b).

My Garfield experience represented a traditional “expert‐meets‐community at a public meeting.” There was an undercurrent of outrage, and a different message would have been greeted with derision, I'm sure. My intention was to increase knowledge and concern. I told them that they had a potentially significant exposure. The message was successful in achieving the objective of people taking the chromium seriously, signing up for a lawsuit, and participating in the biomonitoring program planned by NYU Medical School. I could focus on chromium toxicity and health risks, clarify the inevitable confusion over “hex,” and answer questions about cancer risk and the potential benefits and limitations of a medical monitoring or biomonitoring program. After the lecture, I had no further connection with the community.

### 4.9 Hydrogen sulfide from the Fenimore Landbill—2012–2015

In September 2013, EOHSI received a request for technical assistance from the town of Roxbury (Morris County, NJ). The town was plagued by the rotten egg odor of hydrogen sulfide (H_2_S) emanating from the nearby Fenimore Landfill. Paul Lioy and I visited Roxbury to meet with the town Team, the mayor, manager, engineer, health officer, and attorney. “It stinks” we were told in advance. Indeed, it did. The odor of rotten eggs can be detected and identified even at the low part per billion level. H_2_S is a well‐known, well‐studied gas, lethal at high concentrations, irritating at medium levels, and annoying at low levels. We smelled it as we drove into Roxbury to meet our primary risk communication audience, the Town's leadership “Team.”

The odor had been present for almost a year and was getting worse and more frequent, it seemed. The story made national news (ABC7, 2013). With thinly veiled frustration, the Team gave us the following history which we accepted at face value at the outset, although there were some minor discrepancies in subsequent versions (Gurian, 2014). In October 2012 Hurricane *Sandy* had destroyed over 300,000 homes on the Jersey shore. Without consultation with the town, the NJDEP had designated the long‐inactive Fenimore Landfill, immediately adjacent to the town, to receive the construction debris. Over a period of months more than 10,000 truckloads were transported on town roads to the landfill. Much of this was water‐damaged wallboard. Wallboard is comprised of gypsum, calcium sulfate, which over time decays in a landfill, releasing hydrogen sulfide gas which is responsible for the familiar and unpleasant odor of rotten eggs. Odor complaints began in November 2012. This odor hung over the town, day and night as H_2_S escaped from the landfill, worsening month by month. The community was angry at NJDEP for reopening the landfill, angry at the truckers delivering hundreds of truckloads a day, and most of all at the reputedly crooked landfill operator under indictment, profiting at the town's expense. Involuntary exposure, helplessness, and someone else profiting, were all ingredients for heightening the apparent riskiness of the toxic gas (Slovic et al., 2000). There were odor complaints, health complaints and outrage in the town. For some in nearby houses there was no escape from the odor. Some had already moved away.

Our next audience was town residents invited to a public meeting. Only about two dozen people attended. Before the meeting we had read media accounts indicating the Fenimore landfill story was about crime, corruption, deception, as well as odor. At the meeting we did a lot of listening and asked a lot of questions. We reviewed the health complaint registry, and data generated from the H_2_S monitors. The H_2_S alarms, set to 30 ppb were going off frequently. We subsequently met with an expanded audience of town residents. Risk communication included providing preliminary toxicology information, mostly validating the readily available information that the town had already assembled. Numerous odor complaints were filed each week after *Sandy*. We learned it was not just odor complaints, but irritation symptoms attributable to the toxic gas. There were health effects, worsening of asthma and irritation of eyes, nose, and throat, as well as manifestations of anger and stress and outrage. H_2_S is heavier than air so it moved downhill from the landfill into the town.

Our next step was to assess the hazard and examine the H_2_S levels documented by the monitors. Often the levels were very low for hours and then there would be excursion to high levels, well in the range known to cause symptoms. “It burps,” someone explained. The H_2_S gas was continuously generated and would build up in pockets of debris for hours and then explode its way out. Town residents were vocal about condemning the NJDEP, criticizing town leadership, and insisting that something be done. Some citizens wanted all the debris removed and trucked somewhere else—“10,000 truckloads to Trenton” one person chanted. However, this view was very unpopular with people living along the trucking route. They favored a thicker cap and an extraction system to suck out the H_2_S as it was generated. And, they wanted to hear from us about the health risks. What did all those numbers—detection levels, reference concentrations and minimal risk levels, mean for their health. What did it mean when an H_2_S meter alarm sounded?

I explained how the EPA (EPA, 1993) and ATSDR (ATSDR, 2018) arrived at the different levels (reference concentrations, minimal risk levels), which were considered safe for lifetime exposure or more specifically “likely to be without an appreciable risk of deleterious effects during a lifetime.” (IRIS, 2022) and different levels considered dangerous in the short‐term. The official definitions of “acute,” “subacute,” and “chronic,” were hard to interpret here since the high exposures were intermittent and usually brief. I explained about exposure, concentration, and duration. With one group we examined the Acute Exposure Guideline Levels (AEGL tables), which list dangerous levels for exposures from 10 min up to 8 h (EPA, 2010). Some people were actually unhappy to hear that the levels were not hazardous in the long term, but I acknowledged that they caused stress, and that chronic stress, coupled with the unavoidable sense of helplessness, was both harmful and a risk factor for cardiovascular disease (Dimsdale, 2008).

I did not sense hostility in the audience's questions. We heard that the angriest people had already moved away. Here, people were troubled by the uncertain information, for example, that H_2_S can be fatal and that as the concentration increases, the odor goes away before a fatal level is reached (Guidotti, 2010). This unique feature of H_2_S, olfactory paralysis at high concentrations, proved very troublesome for communication. After all, people explained, they could not always smell it. First it was there and then faded away. Did that signify a dangerous level? It sounded pretty worrisome. This is universally true of odors, not just H_2_S. Our sensory system adapts over minutes or hours. As I thought about how to explain this, I could feel a rabbit‐hole approaching. I changed the subject.

“A lethal level is 100 parts per million,” I explained. Someone responded immediately, “I thought the monitors have registered 100.” Someone else corrected, “100 parts per billion not million on the monitors.” It should be common knowledge that parts per million are larger than parts per billion—but looking at that sentence, it is not intuitively obvious why a billion is not more than a million. Nor would I expect most people in the audience to know immediately that there is a thousand‐fold difference. I did not want to get sidetracked from the message that even the highest level recorded by any monitor thus far was far below a dangerous acute level.

Odor and annoyance had not persuaded NJDEP to make Roxbury a priority, although early in 2013 it had closed the landfill and diverted subsequent debris elsewhere. ATSDR has an acute minimal risk level of 70 ppb for H_2_S. That level was exceeded on the Roxbury monitors on many occasions. The Town Team explained that the NJDEP seemed dismissive about the H_2_S levels, because levels were usually below 100 ppb, a level NJDEP associated with neurologic effects. “If the odors reached Trenton, they would do something about it.” Odors were considered only a nuisance, rather than a toxic endpoint.

Therefore, the NJDEP became another audience for Lioy and me. Whereas we were mostly reassuring to the Team and the town, we took the opposite tack with the State. Subsequent emails and phone discussions emphasized that stress, anger and outrage are significant health endpoints and are risk factors for other diseases (Williams et al., 2000), particularly for people who were already feeling anxious about their toxic exposure (Deschênes et al., 2012). These are not typical health endpoints in risk assessments. But they were key to the risk communication.

DEP was inclined to disregard odor as a significant endpoint because of its subjectivity. But it did recognize the occasional exceedances of the 100 ppb level. They discussed plans for a novel gas extraction‐incineration system and already had plans for a demonstration project. I reiterated that there was a literature indicating that stress and anger were risk factors for other diseases and deserve intervention (Williams et al, 2000). “People can smell rotten eggs at 10 ppb,” I reminded them. Lioy had a more succinct message. “It stinks,” he emphasized. We urged the NJDEP to give the Fenimore H_2_S extraction system high priority. The alternative, total removal, would require finding another disposal site which seemed hopeless (NJDEP, 2014). With some repetition of the “it stinks message,” NJDEP responded appropriately. Within a few weeks, a complex system of large tubes was inserted in different parts of the landfill where H_2_S was escaping. The tubes conducted the gas to a portable incinerator which operated effectively to destroy the H_2_S. There was also an oxidizer component to take care of another irritant gas, sulfur dioxide produced from the incinerator. Once it was running 24/7 odor complaints declined. It was a novel technology and a great success. See illustration in Aun (2021).

The NJDOH reported to the Roxbury Community striking a balance between recognizing the toxic nature of H_2_S and reassuring the community that levels had not been high enough, long enough to cause permanent harm. “Tell that to the people who already moved away,” I wanted to say. The DOH reported that H_2_S levels at the school “are typical background levels associated with proximity to a landfill,” as if typical landfill levels would not be an impediment for learning. Today, building a school on or close to a landfill would be considered a major environmental mistake. We agreed with the town that activating a landfill close to a school as happened at Roxbury had been a major mistake.

There were still voices calling for other remediation options including removal. We encouraged the Town to request an ATSDR health assessment. The ATSDR (2016) report was released several months after our role had wound down. It emphasized that odor complaints were significant endpoints and that continued operation of the extraction/incineration unit was appropriate to limit releases of H_2_S. More specifically it acknowledged that H_2_S concentrations above ATSDR's acute minimal risk level (MRL) of 70 ppb were frequent, and were high enough to cause changes in lung function or headaches in people who are exposed. This was gratifying validation of our message about exacerbating asthma and stress responses to the hateful odor. ATSDR (2016) urged continued operation of the vapor extraction/incineration system “indefinitely.” The system is still operating, with some upgrades, in 2022 (Aun, 2021).

So in conclusion our H_2_S risk communication was staged over an 18‐month period through a series of meetings and email exchanges. We had somewhat opposite messages for our multiple audiences, reassurance for the Town while conveying urgency to the State.

### 4.10 Environmental justice: The unfinished school and community outrage

I am saving the biggest risk communication challenge for last, illustrating the power of outrage (Sandman, 1989) when risk becomes irrelevant. In some cases, we get a gratifying response to our risk communications. Some people sign up or sign on. But in the case of an unnamed New Jersey School, risk communication did not stand a chance.

EOHSI had been invited to evaluate an exposure concern at the site of a half‐completed middle school where construction had been halted. The Community would be considered “burdened” under New Jerseys current Environmental Justice Law (NJDOJ, 2020). Again, Paul Lioy and I were the respondents. We had been apprised of an emerging environmental justice crisis. It had come belatedly to public attention that at least one of the dozens of truckloads of fill had subsequently been identified as containing polyaromatic hydrocarbons (PAH) and polychlorinated biphenyl (PCB) concentrations above the State's acceptability criteria. The exceedances were not trivial, but the risk was. The truckload, if it was the only one, would have been diluted by many other loads. But in any case, the fill was now covered by a 10‐inch thick concrete slab. Risk communicators have a mantra, “no exposure, no risk.” The slab would prevent anyone from being exposed to chemicals in the soil below.

As we surveyed the half‐completed school in the middle of a largely black, residential neighborhood, we could envision truckloads of fill arriving months earlier. The fill was now safely covered by the thick concrete slab of the completed basement. The first floor was nearly complete, and above it rose the steel skeleton of the second floor. Short of a seismic event there would not be any exposure to the fill beneath the slab. We said so to our guide from the school district. After our site visit the school board meeting was very tense. We explained to our audience, the school board, and public attendees, that although there were some bad chemicals under the slab, and someone had made bad decisions, for now and the future the slab was a barrier that would safely block any exposure to the chemicals. Toxicity and exposure would not be an issue for their children and teachers. For me as an outsider there was no toxicologic rationale for digging out the contamination, which required tearing down the structure and breaking up the slab. I confess that I was feeling offended that tearing up the school was being considered, that it was a misuse of environmental regulations and that no risk reduction would be accomplished. I mentioned only the last point.

The rejection was instant, angry, and dismissive. Several audience speakers explained the contentious history between the City and Community, a long racist history. We quickly recognized the outrage. I tried to explain “no exposure, no risk,” but was again dismissed. There rose, instead, an outcry that the toxic building be dismantled, the slab removed, and the offending fill carted away, “use it for a white school,” someone suggested. Our message was shouldered aside, and we heard stories how this was a just another in a long line of insults that the community had endured from the City. Someone told us “probably some inspector was on the take, to let that load get through,”—a clear hit on one of Paul Slovic's criteria for heightened risk perception (Slovic et al., 2000).

The decision was made. The community, or its loudest voices, insisted the structure be torn down, the slab chopped up, and the fill—all of it, carted away to right the grievous wrong or decades of wrongs. After further attempts over several weeks to assuage the district and angry parents, the State grudgingly agreed to dismantle the school structure, remove the concrete slab, and dig up all of the fill, clean or contaminated. This was just another in a long line of insults to a community that had wanted and desperately needed a modern middle school. Yet no one could defend the structure that seemed to be viewed as a monument to crime, corruption, and racism—A monument to be toppled, long before destroying racist statues became fashionable. We were encouraged to shut up. Our risk communication was doomed to failure. We knew enough not to take the dismissal personally, particularly when we were asked by the community to conduct the dust sampling in neighboring homes while the school dismantling and decontamination progressed. This structure, much wanted by the community, had to be sacrificed on the altar of outrage as Peter Sandman (1989) had warned us long ago. As a post‐script, a new school opened on the site, about a decade later.

## 5 DISCUSSION

Although the above cases represent a variety of contexts, audiences, expectations, and directions, there are commonalities as far as a physician toxicologist is concerned. Except for the first two cases, I had the advantage of being a volunteer, engaging with audiences because I had somethings to learn and somethings to offer. The two actual crises (acute events), Three Mile Island (TMI) and Peru, were very different. TMI suffered from no information, misinformation, rapid evolution, and the utility's good luck in defusing the hydrogen bubble. New Jersey made the right decision, not panicking, reassuring citizens, and not recommending evacuation. In retrospect that should have been easy, but at the time it was tense and frightening. We had no frame of reference for evaluating “meltdown” and “nuclear explosion.” In Peru we encountered indigenous beliefs regarding mercury, distrust because of our association with the company, and a lack of useful information linking exposure (measured in homes) with symptoms or outcomes. I very much enjoyed the experience and challenge that the mercury spill afforded, but the lack of information and cooperation was frustrating and robbed the experience of detail.

Some of the risk communication experiences intersected environmental justice (EJ). EPA and DOE developed environmental justice initiatives and priorities taking into account the Executive Order 12898. New Jersey expanded on that in 2021. The unnamed school, of course, was all about environmental justice (EJ). The chromium cases were largely EJ as well, with lower middle class and communities of color overrepresented as immediate neighbors of the historic chromium industries. On the other hand, Maywood and Roxbury were not obviously EJ communities, although SES status within those communities would have influenced the ability to move out temporarily or away permanently.

Asbestos occupied half of my time at the health department and I stayed with it long after leaving the Department, because it is such an important part of the fabric of Environmental and Occupational Medicine, particularly in New Jersey. An entire volume could profitably be devoted to risk communication around asbestos on an international scale, beyond the two cases described here (Gochfeld, 1997). Likewise, chromium occupied much time and energy but was much messier from an epidemiologic perspective, in the sense that epidemiologic data were less stark. In both cases, asbestos and chromium, the industries employed cadres of mercenary scientists and epidemiologists to create confusion and sow seeds of doubt (Michaels, 2008) in the mind of the public and policymakers.

Both the asbestos and chromium stories deal with carcinogens. Much of the history of risk communication focuses on how to explain cancer probabilities, particularly infinitesimally small probabilities, to reassure the community that you have just frightened by talking about their carcinogen exposure. I tried to steer away from probabilities. I found that people who are exposed to carcinogens are more interested in how to end exposure as soon as possible, and then what can be done about their past exposure. They tune out numbers like 1 in 10,000 or 1 in a million. These criteria are arbitrary in the first place, highly, contentious, and the risk calculations are also fraught with uncertainty. Once they know a carcinogen is involved, no one has ever asked me if 4 times 10 to the minus 6 is bad for them or is really “acceptable.”

Some of the cases involved long‐term relationships with communities and others were one‐night stands (Garfield Chromium & Maywood Thorium). These meetings were at the request of lawyers who paid an honorarium to convey a message that clarified or enhanced the riskiness of exposures and encouraged attendees to join a class action. I have provided a disclosure at the beginning of each of these. In the Maywood case it resulted in a 15‐year medical monitoring program which ultimately moved to EOHSI. In Garfield biomonitoring was conducted by colleagues in the Environmental Medicine department of New York University.

Among the long‐term relationships, Roxbury was largely satisfying in that we were able to provide reassurance regarding long term effects, help establish action levels for hydrogen sulfide exposure, and also encourage the NJDEP to take odor complaints and stress as serious environmental outcomes that require intervention. Once NJDEP set the extraction‐incineration in action 24/7 odor complaints declined dramatically. Messages have to be tailored differently for different audiences.

One of the valuable tools for understanding a complex environmental situation is a conceptual site model (CSM), a graphic which identifies sources, pathways, receptors, and risks (Burger et al., 2006). This would have been helpful in several of the cases. It would have been particularly helpful for the mercury and hydrogen sulfide exposures. The CSM illustrates that exposure can be controlled at the source, or by interdicting pathways in various ways, or by influencing the behavior of the receptors (the people).

Communicating risk to an audience is rarely simple, and sometimes frightening. Bringing good news to a community outraged by injustice and seeking redress in court, is unwelcome. Bringing bad news to a community worried more about property values than health, is unwelcome as well. Although there are always realities, the actual facts are seldom known in full at critical points.

Communicators carry baggage, based on their affiliations and mental model of whether life is intrinsically risky or intrinsically safe, whether risks are overstated and over‐feared, or the contrary. The Slovic‐Fischoff dichotomies help explain relative fearfulness: known versus unknown, caused by someone for profit, immediate versus distant, and most importantly, voluntary or not. However, within this framework the same narrative may lead one person to worry and another to enjoy.

There are articles and manuals as well as web pages with important lessons for risk communication (e.g., Cho et al., 2015; Covello et al., 1989; Kasperson, 1986; Weinstein & Sandman, 1993). Before undertaking a risk communication assignment, it is important to realize that the audience(s) know a lot about the context and history of the event and have probably researched the technical aspects as well. Plan to listen and respect their views. What do they want to know? Many risk communication scenarios involve a beleaguered expert facing a hostile outraged audience (Sandman, 1989). I can think of several personal experiences like that, particularly talking to our own University audiences about hazards such as asbestos in buildings, including our own building. Only the *Unfinished School* example given was characterized by outrage. Most of the risk communication activities have been stimulating, even rewarding. I have apparently successfully blocked memories of the most unpleasant ones.

## 6 CONCLUSIONS AND LESSONS LEARNED

These cases and other experiences taught some lessons about what communicators need to know about environmental health events, audiences, risks, and about themselves.

**About the event**

–Know the event, its context and ontogeny? Is it a short time crisis or a chronic condition?
–Is the situation improving or worsening?
–What is the hazard)? What are the potential exposure pathways?
–Who is exposed (now or before or in the future)?
–What do we know or can we know about risk?
–What can be done about it?
–Is there a conceptual site model or can one be developed?
–is there an end or resolution in sight?

**About the audiences**

–Realize that the audience knows much more about the event than you do.
–Know the audiences and their roles and stakes
–Know the audiences and their mental model(s), are they passive or outraged?
–Are they open minded?
–Will they welcome or distrust reassurance?
–Don't say “trust me.”

**About the communicator**

–Know your objective, the “why me and why am I here.” Is your role to lower or heighten fears?
–Know your baggage, does your employment or association strengthen or weaken your authority and trustworthiness?
–Know your own mental model: Do you believe the audience is over‐reacting/under‐reacting?
–How will you answer: “Would you let your mother live here?”

**About the moral context**

–Is this an environmental justice case?
–Is there a clear responsible party?
–Is the exposure voluntary and/or controllable?
–What is the legal context and how does it color risk perception? Is there a crime?

**About the risks**

–Is the likelihood of risk high and certain or very uncertain?
–Is there a cancer risk?
–Is there a non‐cancer/toxic risk?
–Is there are reproductive risk?
–Is there mainly a property value risk?
–Are the risks great?
–Again, do you believe the risk perceptions over, under, or accurately reflect risks?

**About the messaging**

–Do I know enough about any of the above to speak with confidence?
–Would I indeed let my mother live here? Would I let my mother move here?
–Should we let others live here?
–What can be done about it and who needs to do it?

## References

[risa14006-bib-0001] ABC7 . (2013, June 11). Roxbury residents angry over gas from landfill. *ABC Channel 7 News* . https://abc7ny.com/archive/9135699

[risa14006-bib-0002] Annest, J. L. , Pirkle, J. L. , Makuc, D. , Neese, J. W. , Bayse, D. D. , & Kovar, M. G. (1983). Chronological trend in blood lead levels between 1976 and 1980. New England Journal Medicine, 308, 1373–1377. 10.1056/NEJM198306093082301 6188954

[risa14006-bib-0003] Arana‐Zegarra, M. (2009). El caso del derrame de mercurio en Choropampa y loes daños a la salud en la población rural expuesta. Revista Peruana de Medicina Experimental y Salud Publica, 26(1), 113–118. http://www.scielo.org.pe/pdf/rins/v26n1/a19v26n1.pdf [5/8/2022]

[risa14006-bib-0004] Arvai, J. , & River, L. III (eds). (2013). Effective risk communication. (p. 360). Routledge. 10.4324/9780203109861

[risa14006-bib-0005] Asbestos Hazard Emergency Response Act [AHERA]. (1986). Environmental protection agency . https://www.govinfo.gov/content/pkg/USCODE‐2009‐title15/html/USCODE‐2009‐title15‐chap53‐subchapII.htm

[risa14006-bib-0006] ATSDR. (1990). Health assessment for Maywood Chemical Company, Maywood, Bergen County, New Jersey . Agency for Toxic Substances and Disease Research. https://www.state.nj.us/health/ceohs/documents/eohap/haz_sites/bergen/maywood/maywood_chem_co/maywood_pha__7_90.pdf

[risa14006-bib-0007] ATSDR . (2012). Chemical‐specific health consultation for joint EPA/ATSDR national mercury cleanup policy workgroup action levels for elemental mercury spills . Agency for Toxic Substances and Disease Research. https://www.atsdr.cdc.gov/emergency_response/Action_Levels_for_Elemental_Mercury_Spills_2012.pdf

[risa14006-bib-0008] ATSDR. (2016). Health consultation: Evaluation of community exposures and concerns related to Fenimore Landfill, Roxbury Township, New Jersey, May 11, 2016 . Agency for Toxic Substances and Disease Research. https://www.atsdr.cdc.gov/hac/fenimorelandfill_hc_final_may112016_508.pdf [5/8/2022]

[risa14006-bib-0009] ATSDR. (2018). Minimal risk levels (MRLS) . Agency for Toxic Substances and Disease Research. https://www.atsdr.cdc.gov/minimalrisklevels/index.html Originally in 1980, updated 2018

[risa14006-bib-0010] Aun, F. J. (2021). Two more years of burning away stinky gas at Roxbury's Fenimore dump. Tap Into Roxbury, (May 31, 2018). https://www.tapinto.net/towns/roxbury/sections/government/articles/two‐more‐years‐of‐burning‐away‐stinky‐gas‐at‐roxb

[risa14006-bib-0011] Bach, P. B. , Mirkin, J. N. , Oliver, T. K. , Azzoli, C. G. , & Berry, D. (2012). Benefits and harms of CT screening for lung cancer: A systematic review. JAMA, 307(22), 2418–2429. 10.1102/1470-7330.2012.0049 22610500 PMC3709596

[risa14006-bib-0012] Balmes, J. R. (2013). Asbestos and lung cancer: What we know. American Journal of Respiratory and Critical Care Medicine, 188(1). https://www.atsjournals.org/doi/full/10.1164/rccm.201305‐0885ED 10.1164/rccm.201305-0885ED 23815717

[risa14006-bib-0013] Bellinger, D. C. (2008). Very low lead exposures and children's neurodevelopment. Current Opinions in Pediatrics, 20(2), 172–177. 10.1097/MOP.0b013e3282f4f97b 18332714

[risa14006-bib-0014] Black, K. , Gochfeld, M. , Lioy, P. , Fan, Z.‐H. , Yo, C. H. , Jeitner, C. , Hernandez, M. , Einstein, S. A. , & Stern, A. H. (2015). A post‐remediation assessment in Jersey City of the association of hexavalent chromium in house dust and urinary chromium in children. Journal of Exposure Science & Environmental Epidemiology, 25, 616–622. 10.1038/jes.2015.50 26329141

[risa14006-bib-0015] Bostrom, A. , Fischoff, B. , & Morgan, M. G. (1992). Characterizing mental models of hazardous processes: A methodology and an application to radon. Journal of Social Issues, 48(4), 85–100. 10.1111/j.1540-4560.1992.tb01946.x

[risa14006-bib-0016] Bostrom, A. , Hayes, A. L. , & Crosman, K. M. (2019). Efficacy, action, and support for reducing climate change risks. Risk Analysis, 39(4), 805–828. 10.1111/risa.13210. Epub 2018 Oct 12. PMID: 30368853 30368853

[risa14006-bib-0017] Bostrom, A. , Morgan, G. , Fischoff, B. , & Read, D. (1994). What do people know about global climate change? 1. Mental models. Risk Analysis, 14(6), 959–970. 10.1111/j.1539-6924.2010.01448.x PMC6170370 20649942

[risa14006-bib-0018] Brust‐Renck, P. G. , Reyna, V. F. , Corbin, J. C. , Royer, C. E. , & Weldon, R. B. (2015). The role of numeracy in risk communication. In H. Cho , T. Reimer , & K.A. McComas , (Eds) The SAGE handbook of risk communication (pp. 134–145). SAGE books.

[risa14006-bib-0019] Burger, J. , & Gochfeld, M. (1991). Fishing a superfund site: Dissonance and risk perception of environmental hazards by fishermen in Puerto Rico. Risk Analysis, 11(2), 269–277. 10.1111/j.1539-6924.1991.tb00603.x. PMID: 1876726 1876726

[risa14006-bib-0020] Burger, J. , Mayer, H. K. , Greenberg, M. , Powers, C. , Volz, C. D. , & Gochfeld, M. (2006). Conceptual site models as a tool in evaluating ecological health: The case of the department of energy's Amchitka Island nuclear test site. Journal of Toxicology and Environmental Health, Part A, 69(13), 1217–1238. 10.1080/15287390500360232 16754537

[risa14006-bib-0021] Burke, T. , Fagliano, J. , Goldoft, M. , Hazen, R. E. , Iglewicz, R. , & McKee, T. (1991). Chromite ore processing residue in Hudson County, New Jersey. Environmental Health Perspectives, 92, 131–137. 10.1289/ehp.9192131 1935843 PMC1519394

[risa14006-bib-0022] Burke, T. A. , Gray, S. , Krawiec, C. M. , & Paulson, G. (1980). An environmental investigation of clusters of leukemia and Hodgkin's disease in Rutherford, New Jersey. New Jersey Medical Society Journal, 77(4), 259–264. 6929350

[risa14006-bib-0023] California Cable and Telecommunication Associations (CCTA) . (2021). History of cable. https://calcable.org/learn/history‐of‐cable/[ 1/3/2022]

[risa14006-bib-0024] CAO . (2000). Independent commission report on the mercury spill in the province of Cajamarca, Peru . Compliance Advisor Ombudsman of the International Finance Corporation. https://pressroom.ifc.org/all/pages/PressDetail.aspx?ID=19830 [5/8/2022]

[risa14006-bib-0025] CAO . (2006). Exit report: Regarding two complaints filed with the CAO in relation to Minera Yanacocha, Cajamarca Peru . Compliance Advisor Ombudsman of International Finance Corporation https://www.cao‐ombudsman.org/sites/default/files/downloads/Exitreportenglish02‐7‐06‐final.pdf [12/7/2021]

[risa14006-bib-0026] CDC . (1991). Screening. In Preventing lead poisoning in young children: A statement by the Centers for Disease Control—October 1991. Public Health Service, CDC [5/8/2022].

[risa14006-bib-0027] CDC . (2013). Investigating suspected cancer clusters and responding to community concerns . Guidelines from CDC and the Council of State and Territorial Epidemiologists. MMWR Sept 2013. https://www.cdc.gov/mmwr/preview/mmwrhtml/rr6208a1.htm?s_cid=rr6208a1_w 24067663

[risa14006-bib-0028] CDC . (2022). CDC National childhood blood lead surveillance data . Centers for Disease Control and Prevention, Childhood Lead Poisoning Prevention. https://www.cdc.gov/nceh/lead/data/national.htm

[risa14006-bib-0029] Chess, C. , Hance, B. J. , & Sandman, P. M. (1988). Improving dialogue with communities: A short guide for government risk communication . Office of Science & Research, New Jersey Department of Environmental Protection. https://rucore.libraries.rutgers.edu/rutgers‐lib/31712/pdf/1/ [4/20/22]

[risa14006-bib-0030] Chiu, W. A. , Jinot, J. , Scott, C. S. , Makris, S. L. , Cooper, G. S. , Dzubow, R. C. , Bale, A. S. , Evans, M. V. , Guyton, K. Z. , Keshava, N. , Lipscomb, J. C. , Barone, S. , Fox, J. F. , Gwinn, M. R. , Schaum, J. , & Caldwell, J. C. (2013). Human health effects of trichloroethylene: Key findings and scientific issues. Environmental Health Perspectives, 121(3), 303–311. 10.1289/ehp.1205879 23249866 PMC3621199

[risa14006-bib-0031] Cho, H. , Reimer, T. , & McComas, K. A. (2015). The SAGE handbook of risk communication. SAGE books.

[risa14006-bib-0032] Chromium Cleanup Partnership. (2014). Chromium cleanup partnership chronology. Department of Environmental Protection, City of Jersey City, PPG Industries. http://www.chromecleanup.com/history.html

[risa14006-bib-0033] Covello, V. P. , Sandman, P. M. , & Slovic, P. (1988). Risk communication, risk statistics, and risk comparisons: A manual for plant managers. Chemical Manufacturer's Association.

[risa14006-bib-0034] Covello, V. T. (1989). Communicating information about the health risks of radioactive waste: A review of obstacles to public understanding. Bulletin New York Academy of Medicine, 65(4), 467–482. PMC1808557 2684311

[risa14006-bib-0035] Covello, V. T. (2021). Communicating in risk, crisis, and high stress situations: Evidence‐based strategies and practices. The Institute of Electrical and Electronics Engineers [IEEE]. 10.1002/9781119081753

[risa14006-bib-0036] Covello, V. T. , McCallum, D. B. , & Pavlova, M. R. (1989). Effective risk communication: The role and responsibility of government and nongovernment organizations. Springer.

[risa14006-bib-0037] Deschênes, S. S. , Dugas, M. J. , Fracalanza, K. , & Koerpner, N. (2012). The role of anger in generalized anxiety disorder. Cognitive Behavioural Therapy, 41(3), 261–271. 10.1080/16506073.2012.666564 22429207

[risa14006-bib-0038] Dimsdale, J. E. (2008). Psychological stress and cardiovascular disease. Journal American College Cardiology, 51(13), 1237–1246. doi: 10.1016/j.jacc.2007.12.024 PMC2633295 18371552

[risa14006-bib-0039] DOE . (1992). Remedial investigation report for the Maywood Site. Department of Energy. DOE/OR/21949‐337, October 1992.

[risa14006-bib-0200] DOE FUSRAP . (2022) FACT SHEET – Maywood, NJ: Formerly Utilized Sites Remedial Action Program (FUSRAP). Department of Energy. https://www.nan.usace.army.mil/Media/Fact-Sheets/Fact-Sheet-Article-View/Article/487561/fact-sheet-maywood-nj-formerly-utilized-sites-remedial-action-program-fusrap/

[risa14006-bib-0040] Doherty Lyons, S. P. , Bari, S. , Gany, F. , Leng, J. , Duch, T. , Reveille, D. , Morris, J. S. , Hernandez, M. , Nadas, A. , Costa, M. , & Zelikoff, J. T. (2019). Community health perceptions and human environmental exposure to chromium contamination in a small New Jersey City. Preventive Medicine & Community Health, 2(1). 10.15761/pmch.1000122 PMC8188903 34113778

[risa14006-bib-0041] EPA . (1979). Asbestos in schools guidance document. U.S. Environmental Protection Agency.

[risa14006-bib-0042] EPA . (1993). Reference dose (RfD): Description and use in health risk assessments: Background document 1A March 15, 1993. Integrated Risk Information System, https://www.epa.gov/iris/reference‐dose‐rfd‐description‐and‐use‐health‐risk‐assessments

[risa14006-bib-0043] EPA . (2010). Hydrogen sulfide results: AEGL program . U.S. Environmental Protection Agency. https://www.epa.gov/aegl/hydrogen‐sulfide‐results‐aegl‐program

[risa14006-bib-0044] EPA . (2018). EPA needs to re‐evaluate its compliance monitoring priorities for minimizing asbestos risk in schools. Report 18P‐0270 . U.S. Environmental Protection Agency. https://www.epa.gov/sites/default/files/2018‐09/documents/_epaoig_20180917‐18‐p‐0270.pdf [1/12/2022]

[risa14006-bib-0045] EPA . (2021). *Garfield ground water contamination*. Clean‐up Activities. https://cumulis.epa.gov/supercpad/SiteProfiles/index.cfm?fuseaction=second.cleanup&id=0206317#Status [12/27/2021]

[risa14006-bib-0046] EPA . (2022). Superfund: National priorities list (NPL) . U.S. Environmental Protection Agency. https://www.epa.gov/superfund/superfund‐national‐priorities‐list‐npl

[risa14006-bib-0300] Fagin, D. (2013). Toms Rivers: A Story of Science and Salvation. Bantam Books, New York.

[risa14006-bib-0047] Fischoff, B. , Bostrom, A. , & Quadrel, M. J. (1993). Risk perception and risk communication. Annual Review Public Health, 14, 183–203. 10.1146/annurev.pu.14.050193.001151 8323585

[risa14006-bib-0048] Fischoff, B. , Slovic, P. , & Lichtenstein, S. (1977). Knowing with certainty: The appropriateness of extreme confidence. Journal of Experimental Psychology, 3(4), 552–564.

[risa14006-bib-0049] Fischoff, B. , Slovic, P. , Lichtenstein, S. , Read, S. , & Combs, B. (1978). A psychometric study of attitudes towards technological risks and benefits. Policy Sciences, 9, 127–152.

[risa14006-bib-0050] Friends of the Earth. (2015). Thorium and WMD proliferation risks. Friends of the Earth—Australia. Covington. https://www.foe.org.au/anti‐nuclear/issues/nfc/power‐weapons/thorium [1/6/2022]

[risa14006-bib-0051] Gardoni, P. , & Murphy, C. (2014). A scale of risk. Risk Analysis, 34(7), 1208–1227. 10.1111/risa.12150 24372160

[risa14006-bib-0052] Gochfeld, M. (1997). Asbestos hazards: A risk communication challenge. In G.A. Peters & B.J. Peters (Eds.). Asbestos and cancer (Vol 16, pp. 403–423). LEXIS Publishing.

[risa14006-bib-0053] Gochfeld, M. (2003a). Why epidemiology of endocrine disruptors warrants the precautionary principle. Pure & Applied Chemistry, 75(11–12), 2521–2529.

[risa14006-bib-0054] Gochfeld, M. (2003b). Cases of mercury exposure, bioavailability, and absorption. Ecotoxicology & Environmental Safety, 56(1), 174–179. 12915150 10.1016/s0147-6513(03)00060-5

[risa14006-bib-0055] Gochfeld, M. , Bogden, J. D. , & Louria, D. B. (2009). Ethics and principles in medical monitoring of populations exposed to environmental hazards. Journal Occupational & Environmental Medicine, 51(12), 1363–1366. 10.1097/JOM.0b013e3181c75096 PMC4387773 19952784

[risa14006-bib-0056] Gochfeld, M. , & Burger, J. (2011). Disproportionate exposures in environmental justice and other populations: the importance of outliers. American Journal Public Health.101 Suppl(S1:S53‐S63. 10.2105/AJPH_2011_300121 PMC3222496 21551384

[risa14006-bib-0057] Gochfeld, M. , Burger, J. , Kosson, D. , & Brown, K. (2022). An approach for evaluating contaminants of concern and communicating risk to the public using ETTP of Oak Ridge as a case study. Waste Management Proceedings 2022. Waste Management 2022.

[risa14006-bib-0058] Gochfeld, M. , & Goldstein, B. D. (1999). Lessons in environmental health in the twentieth century. Annual Review of Public Health, 20, 35–53. 10.1146/annurev.publhealth.20.1.35 10352848

[risa14006-bib-0059] Gochfeld, M. , & Witmer, C. (1991). A research agenda for the environmental health aspects of chromium. Environmental Health Perspectives, 92, 141–144. https://ehp.niehs.nih.gov/doi/pdf/10.1289/ehp.9192141 1682141 10.1289/ehp.9192141 PMC1519396

[risa14006-bib-0060] Goldman, K. D. , Demissie, K. , DiStefano, D. , Ty, A. , McNally, K. , & Rhoads, G. G. (1998). Childhood lead screening knowledge and results of a New Jersey physician survey. American Journal Preventive Medicine, 15(3), 228–234. 10.1016/s0749-3797(98)00072-5 9791641

[risa14006-bib-0061] Goldstein, B. D. (2005). Advances in risk assessment and risk communication. Annual Review Public Health, 26, 141–163. 10.1146/annurev.publhealth.26.021304.1444 15760284

[risa14006-bib-0062] Goldstein, B. D. , Demak, M. , Northridge, M. , & Wartenberg, D. (1992). Risk to groundlings of death due to airplane accidents: A risk communication tool. Risk Analysis, 12(3), 339–341. 10.1111/j.1539-6924.1992.tb00685.x 1410705

[risa14006-bib-0063] Goldstein, B. D. , Erdal, S. , Burger, J. , & Faustman, E. M. (2000). Stakeholder participation: Experience from the CRESP program. Environmental Epidemiology and Toxicology, 2(2), 103–111.

[risa14006-bib-0064] Greenberg, M. (1993). Proving environmental inequity in siting locally unwanted land uses. RISK: Health, Safety & Environment (1990–2002), 4(3), Article 5. https://scholars.unh.edu/cgi/viewcontent.cgi?referer=&httpsredir=1&article=1137&context=risk

[risa14006-bib-0065] Guidotti, T. L. (2010). Hydrogen sulfide: Advances in understanding human toxicity. International Journal Toxicology, 29(6), 569–581. 10.1177/1091581810384882 21076123

[risa14006-bib-0066] Gurian, S. (2014). Is Sandy debris in Roxbury's Fenimore landfill poisoning town's children, adults? NJ Spotlight News: Energy and Environment. https://www.njspotlightnews.org/2014/07/14‐07‐07‐is‐sandy‐debris‐in‐roxbury‐s‐fenimore‐landfill‐poisoning‐town‐s‐children‐adults/

[risa14006-bib-0067] Halperin, W. , Altman, R. , Stemhagen, A. , Iaci, A. W. , Caldwell, G. , Mason, T. , Bill, J. , Abe, T. , & Clark, J. F. (1980). Epidemiologic investigation of clusters of leukemia and Hodgkin's disease in Rutherford, New Jersey. Journal of the Medical Society of New Jersey, 77, 267–273. 6929351

[risa14006-bib-0068] Hampson, S. E. , Andrews, J. A. , Lee, M. E. , Foster, L. S. , Glasgow, R. E. , & Lichtenstein, E. (1998). Lay understanding of synergistic risk: The case of radon and cigarette smoking. Risk Analysis, 18(3), 343–350. 10.1111/j.1539-6924.1998.tb01300.x 9664729

[risa14006-bib-0069] Henschke, C. I. , McCauley, D. I. , Yankelevitz, D. F. , Naidich, D. P. , McGuinness, G. , Miettinen, O. S. , Libby, D. M. , Pasmantier, M. W. , Koizumi, J. , Altorki, N. K. , & Smith, J. P. (1999). Early lung cancer action project: Overall design and findings from baseline screening. Lancet, 354(9173), 99–105. 10.1016/S0140-6736(99)06093-6 10408484

[risa14006-bib-0070] Honeywell International. (2010). Consent decree regarding sites 79 and 153 South. [1/21/2010].

[risa14006-bib-0071] IRIS. (2022). Integrated risk information system: IRIS toxicity values. Office of Research and Development, Environmental Protection Agency. https://www.epa.gov/iris/basic‐information‐about‐integrated‐risk‐information‐system#:~:text=Reference%20Concentration%20(RfC),in%20EPA's%20noncancer%20health%20assessments

[risa14006-bib-0072] Jacquez, G. M. , Waller, L. A. , Grimson, R. , & Wartenberg, D. (1996). The analysis of disease clusters, Part I: State of the art. Infection Control Hospital Epidemiology, 17(5), 319–327. 10.1086/647301 8727621

[risa14006-bib-0073] Johnson, B. B. (1999). Ethical issues in risk communication continuing the discussion. Risk Analysis, 19(3), 335–348. 10.1023/a:1007084108903 10765408

[risa14006-bib-0074] Kasperson, R. E. (1986). Six propositions on public participation and their relevance for risk communication. Risk Analysis, 6(3), 275–283. 3602500 10.1111/j.1539-6924.1986.tb00219.x

[risa14006-bib-0075] Landrigan, P. J. (1991). The third wave of asbestos disease: Exposure to asbestos in place. Public health control. Introduction. Annals New York Academy. Science, 31(643), xv–xvi. 10.1111/j.1749-6632.1991.tb24438.x 1809120

[risa14006-bib-0076] Leong, M. C. F. , Ho, J.‐C. , Lee, P. C.‐T. , Hokama, T. , Gima, T. , Luo, L. , Sohn, M. , Kim, S. Y. , Kao, S.‐f. , Hsieh, W. A. , Chang, H.‐L. , & Chang, P. W.‐S. (2014). Risk perception of nuclear power plants among university students in Northeast Asia after the Fukushima nuclear disaster. Asia Pacific Journal Public Health, 26(6), 631–641. 10.1177/1010539514532491. Epub 2014 May 1. 24789816

[risa14006-bib-0077] Lippman‐Hand, A. , & Fraser, F. C. (1979). Genetic counseling: Provision and reception of information. American Journal Medical Genetics, 3(2), 113–127. 10.1002/ajmg.1320030202 474624

[risa14006-bib-0078] Lundgren, R. , & McMakin, A. (1998). Risk communication: A handbook for communicating environmental safety and health risks. Battelle Press.

[risa14006-bib-0079] Lundgren, R. , & McMakin, A. (2018). Risk communication: A handbook for communicating environmental safety and health risks (6th ed). Battelle Press. (originally published 1994).

[risa14006-bib-0080] McDonald, T. T. (2013). Chromium removal at Jersey City towers to begin within weeks. The Jersey Journal. https://www.nj.com/hudson/2013/02/chromium_removal_at_jersey_cit.html

[risa14006-bib-0081] Merlo, D. F. , Bruzzone, M. , Bruzzi, P. , Garrone, E. , Puntoni, R. , Maiorana, L. , & Ceppi, M. (2018). Mortality among workers exposed to asbestos at the shipyard of Genoa, Italy: A 55 year follow‐up. Environmental Health, 17, 94. 10.1186/s12940-018-0439-1 30594195 PMC6310930

[risa14006-bib-0082] Michaels, D. (2008). Doubt is their product: How industry's assault on science threatens your health. Oxford University Press.

[risa14006-bib-0083] Moeys, N. (2020, April 02). The village still suffering from Peru mercury spill fallout – after 20 years . *The Guardian*. https://www.theguardian.com/global‐development/2020/apr/02/the‐village‐still‐suffering‐from‐peru‐mercury‐spill‐fallout‐after‐20‐years [12/7/2021]

[risa14006-bib-0084] Morgan, M. G. , Fischhoff, B. , Bostrom, A. , & Atman, C. (2002). Risk communication: A mental models approach. Cambridge University Press. 10.1017/CBO9780511814679

[risa14006-bib-0085] Morgan, M. G. , Henrion, M. , & Small, M. (1990). Uncertainty: A guide to dealing with uncertainty in quantitative risk and policy analysis. Cambridge University Press.

[risa14006-bib-0086] Morgan, M. G. , & Lave, L. (1990). Ethical considerations in risk communication practice and research. Risk Analysis, 10(3), 355–358.

[risa14006-bib-0087] NJDEP . (2010). Hudson county chromate chemical production waste sites. New Jersey Department of Environmental Protection, Site Remediation Division. https://www.nj.gov/dep/srp/siteinfo/chrome/bkgrnd.htm [5//8/2022]

[risa14006-bib-0088] NJDEP . (2014). Fenimore landfill: Long‐term remedy fact sheet (March 28, 2014) . New Jersey Department of Environmental Protection. https://www.nj.gov/dep/fenimore/docs/fact‐sheet‐fenimore‐long‐term‐remedy.pdf [4/20/2022]

[risa14006-bib-0089] NJDEP . (2020). Chrome cleanup partnership . New Jersey Department of Environmental Protection. http://www.chromecleanup.com/history.html

[risa14006-bib-0090] NJDOH ((2008). Analysis of lung cancer incidence near chromium‐contaminated sites in Jersey City: A citizen's guide . New Jersey Department of Health. https://www.nj.gov/dep/dsr/chromium/Analysis%20of%20Lung%20Cancer%20Incidence%20Near%20Chromium%20Contaminated%20Sites%20in%20New%20Jersey_Citizen's%20Guide.pdf [1/16/2022]

[risa14006-bib-0091] NJDOH . (2018). Fact sheet: Environmental health: Asbestos . New Jersey Department of Health. https://www.state.nj.us/health/ceohs/asbestos/asbestos‐faq/

[risa14006-bib-0092] NJDOH . (2019). Childhood lead exposure in New Jersey: Annual report for state fiscal year 2019 . New Jersey Department of Health. https://nj.gov/health/childhoodlead/documents/reports/childhoodlead2019.pdf

[risa14006-bib-0093] NJDOH . (2019). Toms River township childhood cancer investigation . New Jersey Department of Health. https://www.state.nj.us/health/ceohs/environmental‐occupational/hazardous‐waste‐sites/ocean/dovertwp.shtml#1 [5/8/2022]

[risa14006-bib-0094] NJDOH . (2022). Complete health indicator report of cigarette smoking among adults. New Jersey State Health Assessment Data, New Jersey Department of Health. https://www‐doh.state.nj.us/doh‐shad/indicator/complete_profile/CigSmokAdlt.html [1/6/2022]

[risa14006-bib-0095] NJDOJ . (2020). EJ LAW: State's Environmental Justice Law, codified at N.J.S.A ., 13, 1D–157. https://www.nj.gov/dep/ej/docs/njdep‐ao‐2021–25‐environmental‐justice.pdf [4/18/2022]

[risa14006-bib-0096] NJEA . (2016). Districts fail to keep track of asbestos despite law . New Jersey Education Association. https://www.njea.org/districts‐fail‐to‐keep‐track‐of‐asbestos‐despite‐law/ [12/16/2021]

[risa14006-bib-0097] NRC . (1983). Risk assessment in the federal government: Managing the process. National Research Council (NRC), National Academy Press. https://www.nap.edu/catalog/366/risk‐assessment‐in‐the‐federal‐government‐managing‐the‐process [1/12/2022] 25032414

[risa14006-bib-0098] NRC . (1989). *Improving risk communication*. National Academy Press. https://www.nap.edu/catalog/1189/improving‐risk‐communication [1/12/2022]

[risa14006-bib-0099] NRC . (2009). Science and decisions: Enhancing risk assessment . National research council (US) Committee on improving risk analysis approaches used by the U.S. EPA. National Academies Press. https://www.ncbi.nlm.nih.gov/books/NBK214636/ 25009905

[risa14006-bib-0100] Nuttall, W. , & Thomas, P. (2021). Fukushima: Ten years on from the disaster, was Japan's response right? *The Conversation* . https://theconversation.com/fukushima‐ten‐years‐on‐from‐the‐disaster‐was‐japans‐response‐right‐156554

[risa14006-bib-0101] OSHA . (2022). Occupational safety and health standards 1910:1025 Lead . Occupational Safety and Health Administration. https://www.osha.gov/laws‐regs/regulations/standardnumber/1910/1910.1025

[risa14006-bib-0102] O'Toole, T. , & Lescaze, L. (1978, April 09). Medical manhunt seeks link to 16 cancer cases in N.J. Town. *Washington Post* [Rutherford] https://www.washingtonpost.com/archive/politics/1978/04/09/medical‐manhunt‐seeks‐link‐to‐16‐cancer‐cases‐in‐nj‐town/fedfa74a‐11e5‐4007‐81da‐12d0f135c322/

[risa14006-bib-0103] Parker, A. P. , & Banerjee, S. C. (2018). Sharing serious news with cancer patients: Strategies that can help. Cancer Network. https://www.cancernetwork.com/view/sharing‐serious‐news‐cancer‐patients‐strategies‐can‐help PMC11232121 30080918

[risa14006-bib-0104] PCCRARM (1997). Report: Presidential/congressional commission on risk assessment and risk management . https://cfpub.epa.gov/ncea/risk/recordisplay.cfm?deid=55006

[risa14006-bib-0105] Presidential Commission (1979). Report of the President's Commission on the Accident at Three Mile Island. https://www.hsdl.org/?view&did=769775

[risa14006-bib-0106] Rampinelli, C. , Origgi, D. , & Bellomi, M. (2012). Low‐dose CT: Technique, reading methods and image interpretation. Cancer Imaging, 12(3), 548–556. 10.1102/1470-7330.2012.0049 PMC3569671 23400217

[risa14006-bib-0107] Reinhart, R. J. (2019, March 27). Forty years after Three Mile Island, Americans split on nuclear power. *Politics* . https://news.gallup.com/poll/248048/years‐three‐mile‐island‐americans‐split‐nuclear‐power.aspx

[risa14006-bib-0108] Rosen, J. F. (1995). Adverse health effects of lead at low exposure levels: Trends in the management of childhood lead poisoning. Toxicology, 97(1–3), 11–17. 10.1016/0300-483x(94)02963-u 7716776

[risa14006-bib-0109] RWJF (2009). Cigarette smoking prevalence and policies in the 50 states: An era of change – The Robert Wood Johnson foundation impacTeen tobacco chart book. State University at Buffalo. https://tobacconomics.org/uploads/misc/2014/02/chartbook_final060409.pdf [1/6/2022]

[risa14006-bib-0110] Sandman, P. M. (1989). Hazard versus Outrage in the Public Perception of Risk. In V. T. Covello , D. B. McCallum , & M. R. Pavlova (Eds). Effective risk communication: The role and responsibility of government and nongovernment organizations. contemporary issues in risk analysis (Vo. 4) Springer.

[risa14006-bib-0111] Sandman, P. M. , & Paden, M. (1979). At Three Mile Island. Columbia Journalism Review, 43–58. https://www.psandman.com/articles/3‐mile.htm [12/30/2021]

[risa14006-bib-0112] Sandman, P. M. , Sachsman, D. B. , Greenberg, M. R. , & Gochfeld, M. (1987). Environmental risk and the press. Transaction Books.

[risa14006-bib-0113] Selikoff, I. J. , Hammon, E. C. , & Seidman, H. (1979). Mortality experience of insulation workers in the United States and Canada 1943–1976. Annals New York Academy of Science, 330, 91–116. 10.1111/j.1749-6632.1979.tb18711.x.[1/12/2022] 294225

[risa14006-bib-0114] Sholtis, B. J. (2019). As Three Mile Island stops producing electricity, one community braces for change . https://www.witf.org/2019/09/20/as‐three‐mile‐island‐stops‐producing‐electricity‐one‐local‐community‐braces‐for‐change/

[risa14006-bib-0115] Slovic, P. (1987). Perception of Risk. Science, 236(4799), 280–285. 3563507 10.1126/science.3563507

[risa14006-bib-0116] Slovic, P. , Fischhoff, B. , & Lichtenstein, S. (1982). Why study risk perception? Risk Analysis, 2(2), 83–93.

[risa14006-bib-0117] Slovic, P. , Fischoff, B. , & Lichtenstein, S. (2000). Facts and fears: Understanding perceived risk. In P. Slovic (Ed.), The perception of risk (pp. 137–153). Earthscan.

[risa14006-bib-0118] Southwick, R. (2019). Three Mile Island accident was eerily foreshadowed by a Hollywood blockbuster days before . WHYY Website. https://whyy.org/articles/three‐mile‐island‐accident‐was‐eerily‐foreshadowed‐by‐a‐hollywood‐blockbuster‐days‐before/ [NOV 15, 2021]

[risa14006-bib-0119] Stern, A. H. , Gochfeld, M. , & Lioy, P. J. (2013). Two decades of exposure assessment studies on chromate production waste in Jersey City, New Jersey—what we have learned about exposure characterization and its value to public health and remediation. Journal Exposure Science Environmental Epidemiology, 23, 2–12. 10.1038/jes.2012.100 PMC4286875 23131713

[risa14006-bib-0120] Stewart, R. B. , & Stewart, J. B. (2011). Fuel cycle to nowhere: U.S. law and policy on nuclear waste. Vanderbilt University Press.

[risa14006-bib-0121] Taylor, M. (2000). Independent commission appointed to investigate mercury incident near Peruvian mine. International Finance Corporation. https://pressroom.ifc.org/all/pages/PressDetail.aspx?ID=19812

[risa14006-bib-0122] UNEP (United Nations Environmental Programme) . (2013). The mercury issue . http://mddconsortium.org/wp‐content/uploads/2014/11/PNUMA‐2013‐The‐Mercury‐Issue.pdf

[risa14006-bib-0123] United Nations . (2021). Gold production by Country 2021 . https://worldpopulationreview.com/country‐rankings/gold‐production‐by‐country

[risa14006-bib-0124] Wartenberg, D. , & Greenberg, M. (1993). Solving the cluster puzzle: Clues to follow and pitfalls to avoid. Statistics in Medicine, 12(19‐20), 1763–1770. 10.1002/sim.4780121905 8272659

[risa14006-bib-0125] Washington Post . (1979). *What happened: Crisis at Three Mile Island*. *Washington Post*, March 1979. https://www.washingtonpost.com/wp‐srv/national/longterm/tmi/whathappened.htm

[risa14006-bib-0126] Weinstein, N. D. , & Sandman, P. M. (1993). Some criteria for evaluating risk messages. Risk Analysis, 13(1), 103–114. 10.1111/j.1539-6924.1993.tb00733.x

[risa14006-bib-0127] Weinstein, N. D. , Sandman, P. M. , & Hallman, W. K. (1994). Testing a visual display to explain small probabilities. Risk Analysis, 14(6), 895–896. 10.1111/j.1539-6924.1994.tb00053.x 7531358

[risa14006-bib-0128] Weinstein, N. D. , Kolb, K. , & Goldstein, B. D. (1996). Using time intervals between expected events to communicate risk magnitudes. Risk Analysis, 16(3), 305–308. 10.1111/j.1539-6924.1996.tb01464.x 8693157

[risa14006-bib-0129] Welling, R. , Beaumont, J. K. , Petersen, S. J. , Alexeeff, G. V. , & Steinmaus, C. (2015). Chromium VI and stomach cancer: A meta‐analysis of the current epidemiological evidence. Occupational & Environmental Medicine, 72(2), 151–159. 10.1136/oemed-2014-102178 25231674

[risa14006-bib-0130] Williams, J. E. , Paton, C. C. , Siegler, I. C. , Eigenbrodt, M. L. , Nieto, F. J. , & Tyroler, H. A. (2000). Anger proneness predicts coronary heart disease risk: Prospective analysis from the atherosclerosis risk in communities (ARIC) study. Circulation, 101, 2034–2039. 10790343 10.1161/01.cir.101.17.2034

[risa14006-bib-0131] World Nuclear Association . (2020, April). Three Mile Island accident . http://www.world‐nuclear.org/information‐library/safety‐and‐security/safety‐of‐plants/three‐mile‐island‐accident.aspx

[risa14006-bib-0132] Yard, Y. E. , Horton, J. , Schier, J. G. , Caldwell, K. , Sanchez, C. , Lewis, L. , & Gastaňaga, C. (2012). Mercury exposure among artisanal gold miners in Madre de Dios, Peru: A cross‐sectional study. Journal Medical Toxicology, 8(4), 441–448. 10.5696/2156-9614-10.26.200613 PMC3550269 22926732

